# Sustained intestinal epithelial monolayer wound closure after transient application of a FAK-activating small molecule

**DOI:** 10.1371/journal.pone.0304010

**Published:** 2024-08-16

**Authors:** Sema Oncel, Qinggang Wang, Ahmed Adham R. Elsayed, Emilie E. Vomhof-DeKrey, Nicholas D. Brown, Mikhail Y. Golovko, Svetlana A. Golovko, Ricardo Gallardo-Macias, Vadim J. Gurvich, Marc D. Basson

**Affiliations:** 1 Department of Biomedical Sciences, University of North Dakota School of Medicine & Health Sciences, Grand Forks, North Dakota, United States of America; 2 Department of Surgery, University of North Dakota School of Medicine & Health Sciences, Grand Forks, North Dakota, United States of America; 3 Department of Anatomy and Neurobiology, Northeast Ohio Medical University, Rootstown, Ohio, United States of America; 4 Department of Pathology, University of North Dakota School of Medicine & Health Sciences, Grand Forks, North Dakota, United States of America; 5 Institute for Therapeutics Discovery and Development and Department of Medicinal Chemistry, College of Pharmacy, University of Minnesota, Minneapolis, Minnesota, United States of America; 6 Department of Surgery, Northeast Ohio Medical University, Rootstown, Ohio, United States of America; 7 University Hospitals-NEOMED Scholar, Cleveland, Ohio, United States of America; NCMLS, Radboud University Nijmegen Medical Center, NETHERLANDS, KINGDOM OF THE

## Abstract

M64HCl, which has drug-like properties, is a water-soluble Focal Adhesion Kinase (FAK) activator that promotes murine mucosal healing after ischemic or NSAID-induced injury. Since M64HCl has a short plasma half-life in vivo (less than two hours), it has been administered as a continuous infusion with osmotic minipumps in previous animal studies. However, the effects of more transient exposure to M64HCl on monolayer wound closure remained unclear. Herein, we compared the effects of shorter M64HCl treatment in vitro to continuous treatment for 24 hours on monolayer wound closure. We then investigated how long FAK activation and downstream ERK1/2 activation persist after two hours of M64HCl treatment in Caco-2 cells. M64HCl concentrations immediately after washing measured by mass spectrometry confirmed that M64HCl had been completely removed from the medium while intracellular concentrations had been reduced by 95%. Three-hour and four-hour M64HCl (100 nM) treatment promoted epithelial sheet migration over 24 hours similar to continuous 24-hour exposure. 100nM M64HCl did not increase cell number. Exposing cells twice with 2-hr exposures of M64HCl during a 24-hour period had a similar effect. Both FAK inhibitor PF-573228 (10 μM) and ERK kinase (MEK) inhibitor PD98059 (20 μM) reduced basal wound closure in the absence of M64HCl, and each completely prevented any stimulation of wound closure by M64HCl. Rho kinase inhibitor Y-27632 (20 μM) stimulated Caco-2 monolayer wound closure but no further increase was seen with M64HCl in the presence of Y-27632. M64HCl (100 nM) treatment for 3 hours stimulated Rho kinase activity. M64HCl decreased F-actin in Caco-2 cells. Furthermore, a two-hour treatment with M64HCl (100 nM) stimulated sustained FAK activation and ERK1/2 activation for up to 16 and hours 24 hours, respectively. These results suggest that transient M64HCl treatment promotes prolonged intestinal epithelial monolayer wound closure by stimulating sustained activation of the FAK/ERK1/2 pathway. Such molecules may be useful to promote gastrointestinal mucosal repair even with a relatively short half-life.

## Introduction

Gastrointestinal (GI) ulcers are common worldwide. High levels or extensive exposure to noxious agents such as gastric acid, pepsin, medications like nonsteroidal anti-inflammatory drugs (NSAIDs), or inflammation caused by inflammatory bowel disease may cause mucosal injury in the GI tract. The global prevalence of peptic ulcer disease was approximately 8.09 million in 2019 [1]. In the US alone, from 2007 to 2016, the pediatric prevalence of inflammatory bowel disease (IBD) overall increased by 133% whereas the prevalence of adult IBD increased by 123% [2]. Conventional therapy for GI mucosal lesions varies and is directed at neutralizing their cause. This may include proton pump inhibitors (PPIs), histamine-2 receptor antagonists (H2-antagonists), antibiotics, corticosteroids, or other anti-inflammatory agents depending upon the disease. Although such medications reduce the symptoms of GI pathology by allowing the mucosa to heal with less ongoing injury, none of them directly promotes mucosal healing. This represents an important therapeutic lacuna. In addition, we now know that PPIs, which ameliorate upper GI injury from NSAIDs, actually worsen NSAID distal enteropathy by suppressing gastric acid secretion and thus changing the enteric microbiome [3, 4]. Therefore, there is also an immediate need for a therapeutic agent that directly promotes both upper and lower GI healing.

Cell migration is a complex and dynamic multi-step process that involves the assembly and disassembly of focal adhesions (FAs). Focal adhesion kinase (FAK), a non-receptor protein kinase, plays a critical role in the regulation of FAs. FAK is a 125 kDa protein comprised of an N-terminal FERM domain, a central kinase domain, a C-terminal FAT domain, and two linker domains with three PR regions. In an inactive state in the cytosol, there is an interaction between the FAK-FERM and FAK-Kinase domain which prevents FAK autophosphorylation at Y397 [5]. Previous studies identified a commercially available small molecule, ZINC40099027, that activates FAK in vitro [6–8] by acting allosterically on the 35 kDa FAK kinase domain [9], stimulates monolayer wound closure in diverse gastrointestinal epithelial cells at concentrations as low as 10 nM, and promotes mucosal wound healing in rodent gastrointestinal injury models [7, 8]. The signaling pathway for ZN27-induced monolayer wound closure in Caco-2 cells begins with cytosolic activation of FAK, distinct from classical models of FAK activation in the FA, followed by translocation of this activated FAK to the membrane, where its downstream substrate Extracellular Regulated Kinase (ERK) is phosphorylated, leading to FA turnover and epithelial sheet migration [10]. We previously described additional novel small molecule FAK activators that stimulate monolayer wound closure at a concentration as low as 100 pM and promote mucosal wound healing in rodent ischemic small bowel injury [11]. However, both ZN27 and the second-generation small molecules initially studied are non-polar, requiring DMSO-solubilization and potentially accruing DMSO toxicity. We recently synthesized a novel drug-like water-soluble small molecule M64HCl that activates FAK and induces epithelial sheet migration in Caco-2 cells and promotes epithelial restitution in intestinal mucosal healing in mice without obvious toxicity [12]. The effects of M64HCl on Caco-2 monolayer wound closure were preserved when proliferation was blocked by hydroxyurea [12].

M64HCl has promising drug-like properties and a highly favorable therapeutic ratio [12]. However, M64HCl, like the other small molecule FAK activators, so far described, has a relatively short half-life that might require impractically frequent dosing in clinical use. Therefore, the aim of this study was to investigate the span of the motogenic effect of M64HCl after relatively short treatments to evaluate whether a relatively shorter duration exposure to such small molecule FAK activators can have a sustained effect even without continuous treatment.

## Materials and methods

### Reagents

Dulbecco’s modified Eagle’s medium (DMEM) (#25–500) was from Genesee Scientific (San Diego, CA, USA). 0.05% Trypsin-EDTA (#25–300) and Pierce bicinchoninic acid (BCA) Protein Assay Kit (#23225) were obtained from Thermo Fisher Scientific (Waltham, MA, USA). Human transferrin (#10652202001) was obtained from Roche Applied Science (Mannheim, Germany). IRDye conjugated secondary antibodies the anti-rabbit IRDye 680 (#925-68073) and anti-mouse IRDye 800 (#925-32213) were from LI-COR Biosciences (Lincoln, NE, USA). FAK-Tyr-397 antibody (#ab81298) was from Abcam (San Francisco, CA, USA). We also used antibodies to total FAK (Anti-FAK, clone 4.47, #05–537) from EMD Millipore (Temecula, CA, USA). Antibodies to Erk1/2 T-202/Y-204 (#4370), and ERK1/2 (#4696) were from Cell Signaling (Danvers, MA, USA). Collagen type I (#C8919) was obtained from Sigma-Aldrich (St. Louis, MO, USA). The RhoA kinase inhibitor InsolutionTM Y-27632 (#688000) was from MilliporeSigma and was used at a working concentration of 20μM. FAK inhibitor PF573228 (#s2013) was from Selleck Chem (Houston, TX, USA), and was used at a working concentration of 10 μM. ERK kinase (MEK) inhibitor PD98059 (#S1177) was from Thermofisher Scientific and was used at a working concentration of 10 μM. Rho kinase (ROCK) Activity Assay kit (STA-416) was from Cell Biolabs(San Diego, CA, USA).

### Cell culture

Human Caco-2 cells were obtained from American Tissue Culture Collection (ATCC) (Manassas, VA) and maintained at 37°C with 8% CO_2_ in Dulbecco’s modified Eagle medium (DMEM) supplemented with 4.5 g/L D-glucose, 4 mM glutamine, 1 mM sodium pyruvate, 100 U/ml penicillin, 100 μg/ml streptomycin, 10 μg/ml transferrin, 10 mM HEPES pH 7.4, 3.7 g/L NaHCO_3_ and supplemented with 10% fetal bovine serum. Unless otherwise specified, all experiments described here were conducted in cells in this medium, including 10% fetal bovine serum, with the addition of appropriate small amounts of water or DMSO as needed to solubilize experimental agents.

### Ultra-performance liquid chromatography-mass spectrometry (UPLC-MS) analysis for M64HCl concentrations in cells and media

After a two-hour M64HCl treatment, the M64HCl-containing medium was removed, and cells were washed with phosphate-buffered saline (PBS) three times for one minute each, before adding normal cell culture medium. After one hour incubation in a normal culture medium to permit equilibration, cells and media samples (500 μL of cells homogenate and 20 μL of media) were homogenized in methanol (80% final concentration) by sonication. Precipitated proteins were separated by centrifugation at 2,000 xg for 10 min, and 10 μL of supernatant was injected into the UPLC-MS/MS system for analysis. Precipitated proteins were incubated with 1 mL 0.2 M KOH per tube overnight in a water bath at 60°C. Total protein concentrations were measured by using the Pierce bicinchoninic acid (BCA) protein assay kit with a bovine serum albumin standard containing 0.2 M KOH. Then, M64HCl concentrations within cells measured by mass spectrometry were normalized to the total protein concentration measured by the Pierce BCA protein assay kit, making the assumption based upon the literature that the total protein concentration represents approximately 15% of wet cell weight [13, 14]. UPLC separation was achieved using Waters Acquity I Class UPLC system (Waters, Milford, MA, USA) on Waters ACQUITY UPLC HSS T3 column (1.8 μM, 100 Å pore diameter, 2.1 × 150 mm; Waters) with an ACQUITY UPLC HSS T3 precolumn (1.8 μM, 100 Å pore diameter, 2.1 × 5 mm; Waters) heated at 55°C. We used a linear gradient of solvents A (0.1% formic acid in water) and B (0.1% formic acid in acetonitrile) at 0.3 mL/min. At 1 min of separation, initial %B was increased from 1% to 25% during 1 min, and at 2.1 min—to 90% during 1.9 min. At 5 min, solvent B was returned to 1% and allowed 4 min for equilibration between injections. MS/MS analysis was performed on Waters Xevo TQ-S triple quadrupole mass spectrometer (Waters, Milford, MA, USA) using multiple reaction monitoring mode. The MS was operated in a positive ESI mode. The following mass transitions (with collision energies (CE) indicated in parentheses, V) were used: 438.2/273.1 for quantification; 438.2/192.1 and 438.2/149.2 for analyte confirmation. The UPLC-MS/MS system was controlled by MassLynx V4.1. Quantification was performed against an M64HCl external standard using a generated response curve.

### Crystal violet assay

A crystal violet-based assay was used to count cells in order to assess cell proliferation. 200,00 cells were seeded per well in 12 well plates. At 37°C in 8% CO2, the seeded cells were allowed to adhere for 24 hours. Half the wells in the 12-well plate were treated with M64HCl to a final concentration of 100nM in standard cell culture medium and the other half with equal amounts of distilled water (as a vehicle control) in standard cell culture medium. The cells were incubated for 72 hours and then washed 3 times with distilled water. At room temperature, the cells were stained with crystal violet for 30 minutes, then washed 3 times with distilled water before air drying for 24 hours. Methanol was then used to solubilize the cells and crystal violet absorbance was measured with an Epoch microplate reader (BioTek Instruments Inc, Winooski, VT, USA) at 570 nm. The crystal violet absorbance in each well for both the M64HCl-treated and the control cells was normalized to the mean of the control cells in each plate and multiplied by 100 to calculate the percentage of crystal violet absorbance.

### Wound closure

Caco-2 cells were seeded at 80% confluence into 6 well plates pre-coated with type-I collagen [7, 15]. When the cells reached 100% confluence (48 hours after seeding), they were wounded with 200 μl non-barrier autoclaved pipette tips. The stock solution for any required treatment was prepared at 1000-fold higher than the final treatment concentration. The final concentration of each treatment was prepared in 5ml of Caco-2 medium, adding either 5ul of stock solution or 5ul of water vehicle. DMSO was dissolved similarly into a stock solution and treated similarly so that the final DMSO concentration was identical in all wells when a DMSO vehicle was required.

In the first experiment, Caco-2 cells were wounded, and 0-hour images were taken for each monolayer. Then, Caco-2 cells were treated with either M64HCl (100 nM) for 1, 2, 3, 4, and 24 hours or with a water vehicle control. After the indicated incubation time with M64HCl, the M64HCl-containing medium was removed, and cells were washed with PBS three times for one minute each, before adding normal cell culture medium. The cells were then incubated at 37°C until images were taken of the monolayer wounds at 24 hours. In the second experiment, Caco-2 cells were wounded, and 0-hour images were taken for each monolayer. The first two groups of monolayers were treated with either M64HCl (100 nM) or a water vehicle control for 24 hours. Groups 3 and 4 were treated with either M64HCl (100 nM) or a water vehicle control for 2 hours. After the two-hour incubation, the culture medium was removed, and cells were similarly washed with PBS three times before being incubated with a normal medium at 37°C for 10 hours. Then, the cells were treated again for two more hours with either M64HCl (100 nM) or a water vehicle control. After this second two-hour incubation, the medium was again removed and cells were similarly washed with PBS three times, before being incubated with normal media at 37°C for 10 more hours. Then, 24-hour images were taken for all the conditions. Wound images were captured using an inverted light microscope (OLYMPUS CK2, Center Valley, PA) at 0 hours and 24 hours after wounding. Wound areas were measured with Fiji software, and the percentage of wound closure values was calculated in Excel.

### Flow cytometry

Caco2 cells were trypsinized and washed with PBS then centrifuged at 1500rpm for 5 min. Cells were fixed and permeabilized with the FoxP3/Transcription factor fixation/permeabilization kit from eBioscience (San Diego, CA, USA). Cells were immunostained with mouse monoclonal F-actin antibody (#ab130935, Abcam, San Francisco, CA, USA) at a 1:50 dilution for 30 min at room temperature in the dark with gentle shaking. Cells were washed with 150ul 1x permeabilization buffer and centrifuged at 1500rpm for 5 min. Secondary antibody staining was with Goat anti-rabbit IgG A647 (#ab150079, Abcam, San Francisco, CA, USA) at 1:2000 dilution for 30 min at 4oC. Cells were washed 2x with 150ul 1x permeabilization buffer and centrifuged at 1500rpm for 5 min. Samples were analyzed on a BD Symphony flow cytometer (BD, San Jose, CA, USA) and analyzed with FlowJo software (TreeStar, Ashland OR, USA).

### Rho kinase activity assay

Caco-2 cells were seeded in a 6-well plate for 48 hours to reach 85% confluence. Cells were then treated with either water vehicle or M64HCl at 100 nM for 3 hours. 90ul of diluted active ROCK-II positive control or cells lysate samples were loaded into the wells of the substrate plate respectively, and the manufacturer’s assay protocol was followed, with measurement at 450 nm as the primary wavelength of absorbance.

### Immunofluorescence

Caco2 cells were seeded at 50,000 cells per Nunc Lab-Tek II CC^2^ (Thermofisher Scientific) chamber slide well. When cells were 80% confluent, cells were treated with 100 nM M64HCl or water vehicle control for 24 hours. For scratch wounds, cells were 100% confluent, then a scratch wound was made, and media was replaced with 100 nM M64HCl or water vehicle control for 24 hours. Media was removed from each well and cells were fixed with 4% formaldehyde for 10 min at room temperature. The formaldehyde solution was discarded, and wells were washed 3 times with PBS. Cells were permeabilized with 0.1% Triton X-100 in PBS for 15 minutes. Wells were washed 2 times with PBS, then 200ul of 1x Alexa Fluor 647 Phalloidin (Thermofisher Scientific, cat# A22287) with 1% BSA was applied and incubated at room temperature in the dark for 45 minutes. Wells were washed 2 times with PBS and then well slides were removed, and slides were mounted with ProLong Diamond Anifade Mountant with DAPI (Thermofisher Scientific) and covered with a coverslip. Slides were allowed to cure for at least 24 hours. Slides were examined with a Leica DMi8 Stellaris at the UND Imaging Core facility or with an Olympus FV3000 confocal microscope at the UND Pathology Microscopy Core. Fiji ImageJ (NIH) was used to determine mean fluorescence and the plot profile fluorescence over the length of a selected rectangle region from 8.701um from the migrating edge to 100 um into the migrating sheet. Fraction of maximum fluorescence was calculated as previously described by Patel [16].

### Western blotting

To study FAK activation and downstream signaling pathways in adherent migrating cells, Caco-2 cells were sparsely (3500 cells/cm2) seeded to create islands of migrating cells on type-I collagen pre-coated 150mm bacteriologic plastic dishes using ELISA coating buffer as previously described [16]. At 50–60% confluence, cells were treated with 100 nM M64HCl or water vehicle control for two hours at 37°C in 8% CO2. After two hours of incubation, the treated medium was removed, and the cells were similarly washed with PBS three times. Then, cells were incubated with a normal medium at 37°C until harvesting at the indicated time points after the initiation of the two-hour M64HCl treatment. Caco-2 cells were lysed with 80 μL of protein lysis buffer (50 mM Tris, 150 mM NaCl, 1 mM EDTA, 1 mM EGTA, 1% Triton-X-100, 1% deoxycholic acid, 0.1% SDS, 10% glycerol, and protease and phosphatase inhibitors) to extract protein. Protein concentration was estimated by BCA assay (Thermo Fisher, Waltham, MA, USA). 40 μg of proteins (20 μL of protein lysate) were loaded per lane onto 10% SDS-PAGE gels for resolution and transferred onto nitrocellulose membranes as previously described [17]. In this second western blot experiment, the cells were treated for 24 hours with 100nM M64HCl or water vehicle control or 10μM FAK inhibitor PF573228 only or 10μM PF573228+100nM M64HCl or 10μM ERK kinase (MEK) inhibitor PD98059 only or 10μM PD98059+ 100nM M64HCl. We then conducted an additional series of experiments confirming the effects of PF573228 and PD98059 on ERK activation after one-hour treatment. Membranes were blotted with antibody to Y-397-phosphorylated FAK (1:1000) and T-202/Y-204-phosphorylated ERK1/2 (1:2000). Antibody to total FAK (1:1000) and total ERK (1:2000) served as a loading control after stripping and reprobing. Western blots were performed, and images were detected by the LICOR—Odyssey-Fc imaging system (LI-COR Biosciences, Lincon, NE, USA). Densitometry was conducted on exposures within the linear range.

### Statistical analysis

Data are depicted as mean ± standard error of the mean. Data from at least three independent experiments were normalized to the vehicle water values. Results were compared by one-way analysis of variance (ANOVA) followed by a post hoc test (Sidak’s Multiple Comparison Test) seeking 95% confidence.

## Results

### Confirmation that PBS washes effectively remove M64HCl from media and 95% from within Caco-2 cells

**Fig 1:**
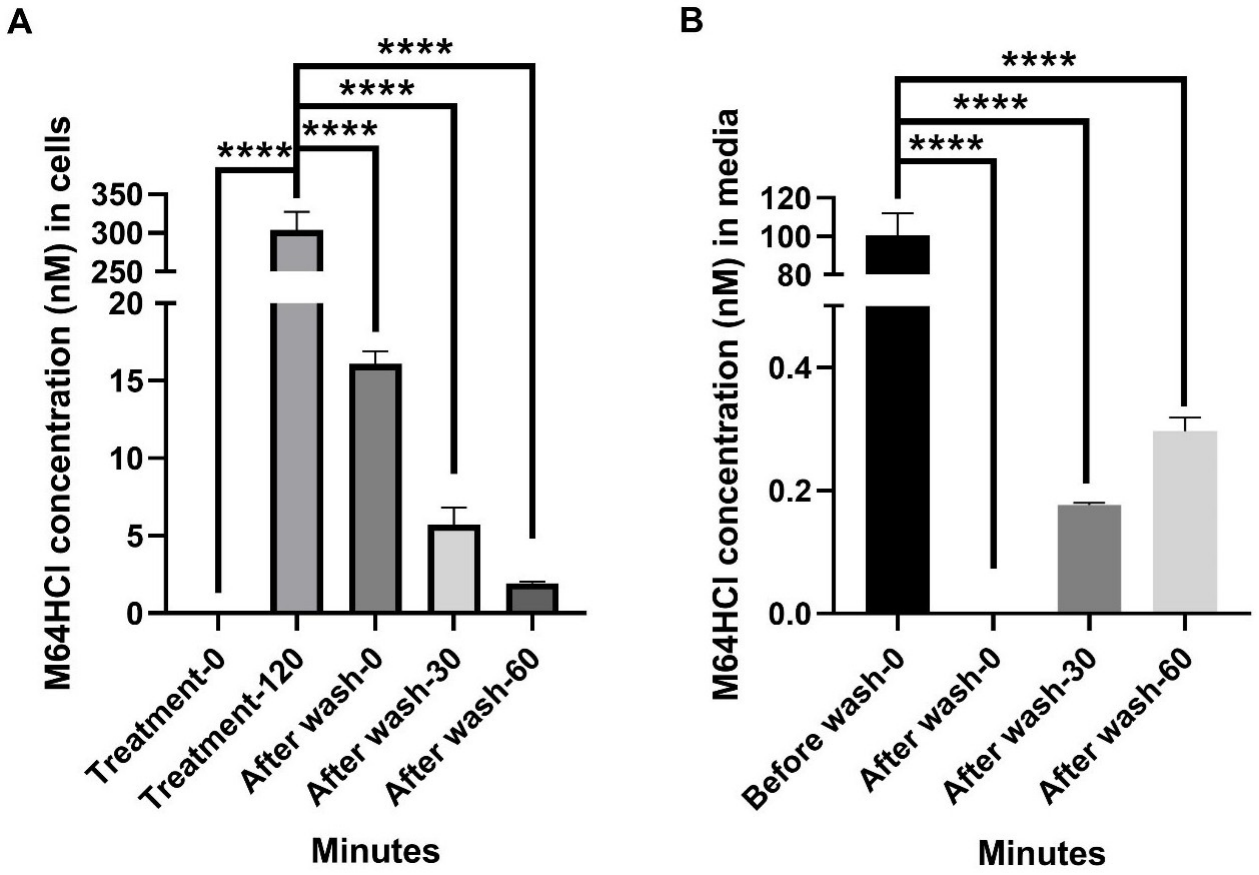
M64HCl concentrations in the media and within the cells before and after washing. (A) M64HCl concentrations within the Caco-2 cells before treatment, two hours after treatment, and 0, 30, and 60 minutes after washing. (B) M64HCl concentrations in the media before and at 0, 30, and 60 minutes after washing. (n = 3–6, ****p < 0.0001).

We began by measuring the level of extracellular and intracellular M64HCl concentrations after a two-hour incubation with M64HCl (100 nM). After a two-hour M64HCl (100 nM) treatment, M64HCl concentrations reached 303.6 ± 23.7 nM (n = 3, p < 0.0001 compared with intracellular measurements prior to treatment) within the cells (Fig 1A). We confirmed the adequacy of our washing steps after treatment by measuring M64HCl concentrations within the cells and in the medium after washing. Washing removed 95% of the intracellular M64HCl, reducing the M64HCl concentration to 16.1 ± 0.8 nM (n = 3, p < 0.0001 compared with the intracellular concentration before washing) immediately after washing (Fig 1A). This gradually trended down further over the next hour, reaching 1.9 ± 0.1 nM (n = 3, p < 0.0001 compared with the intracellular concentration before washing) at 60 minutes after washing (Fig 1A). M64HCl was undetectable in media immediately after washing (0 min) and then gradually trended upward, reaching 0.299 ± 0.023 pM one hour after washing (n = 3, p < 0.0001 compared with the medium concentration prior to washing) (Fig 1B).

### M64HCl promoted prolonged monolayer wound closure in Caco-2 cells

**Fig 2:**
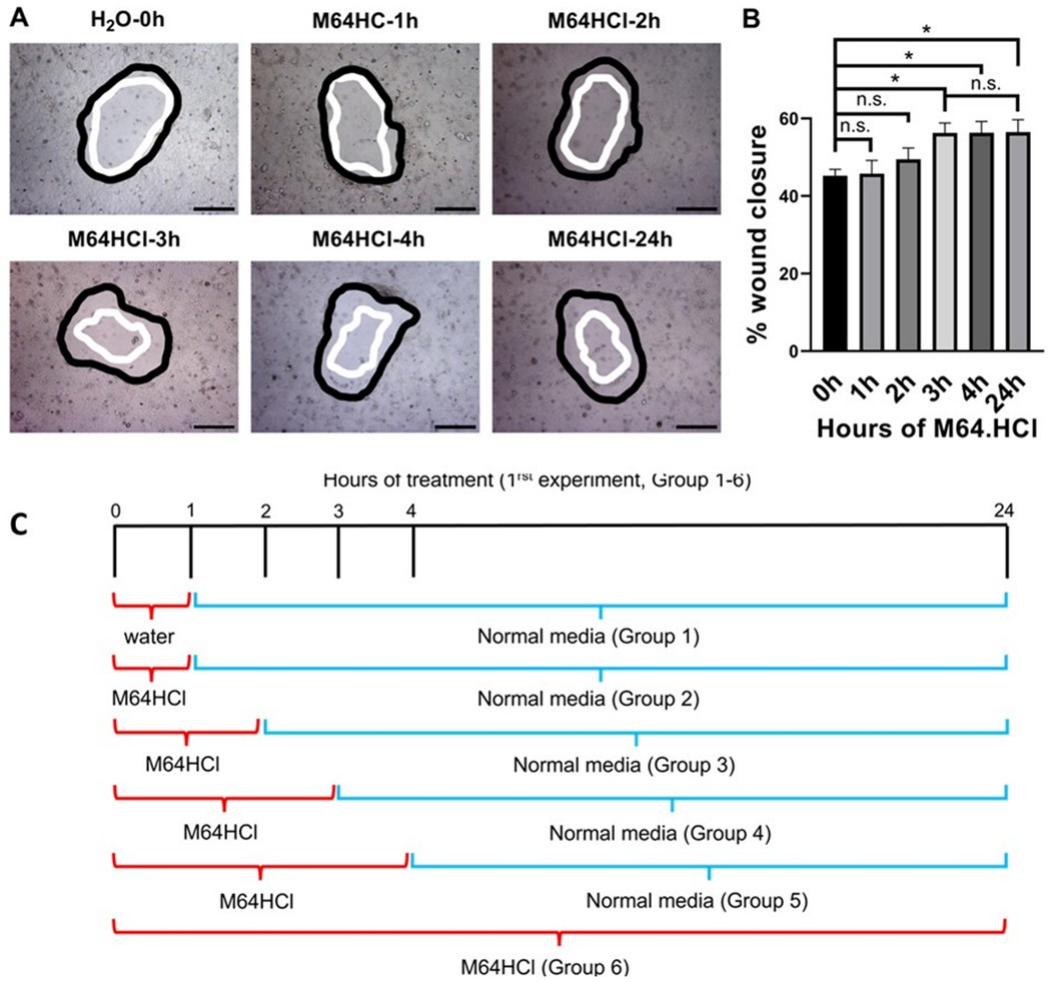
Three- and four-hour treatments of M64HCl stimulated prolonged epithelial monolayer wound closure in Caco-2 cells. (A) Typical wound images for Caco-2 cells treated with H_2_O, M64HCl (100nM) for 1h, 2h, 3h, 4h, and 24h at 0- and 24-hour time points. All images are 4x magnification. Scale bar: 500 μm. (B) M64HCl at 100 nM accelerates circular wound closure in Caco-2 cell monolayers on collagen with 3h, 4h, and 24h treatments (n = 16–24, pooled from 5 separate studies with similar results, *p < 0.05, n.s.: not significant). Black represents the circumference of the 0-hour wounds; white represents the circumference of the wounds 24 hours later. (C) Monolayer wound closure experimental design.

Compared with the water vehicle control, incubation with 100 nM M64HCl for 1 hour (n = 16, p > 0.05) and 2 hours (n = 24, p > 0.05) did not stimulate Caco-2 monolayer wound closure when measured at 24 hours (Fig 2A and 2B). However, 3-hour, 4-hour, and 24-hour incubations with 100 nM M64HCl increased the span of monolayer wound closure at 24 hours in Caco-2 cells by 24.4 ± 5.6% (n = 16, p < 0.05), 24.6 ± 6.4% (n = 16, p < 0.05), and 24.9 ± 7.1% (n = 16, p < 0.05), respectively, in comparison to water vehicle control (n = 24) (Fig 2A and 2B). Moreover, the acceleration of monolayer wound closure by three- or four-hour treatment with M64HCl did not differ from that achieved by a sustained application of M64HCl for 24 hours (Fig 2A and 2B).

**Fig 3:**
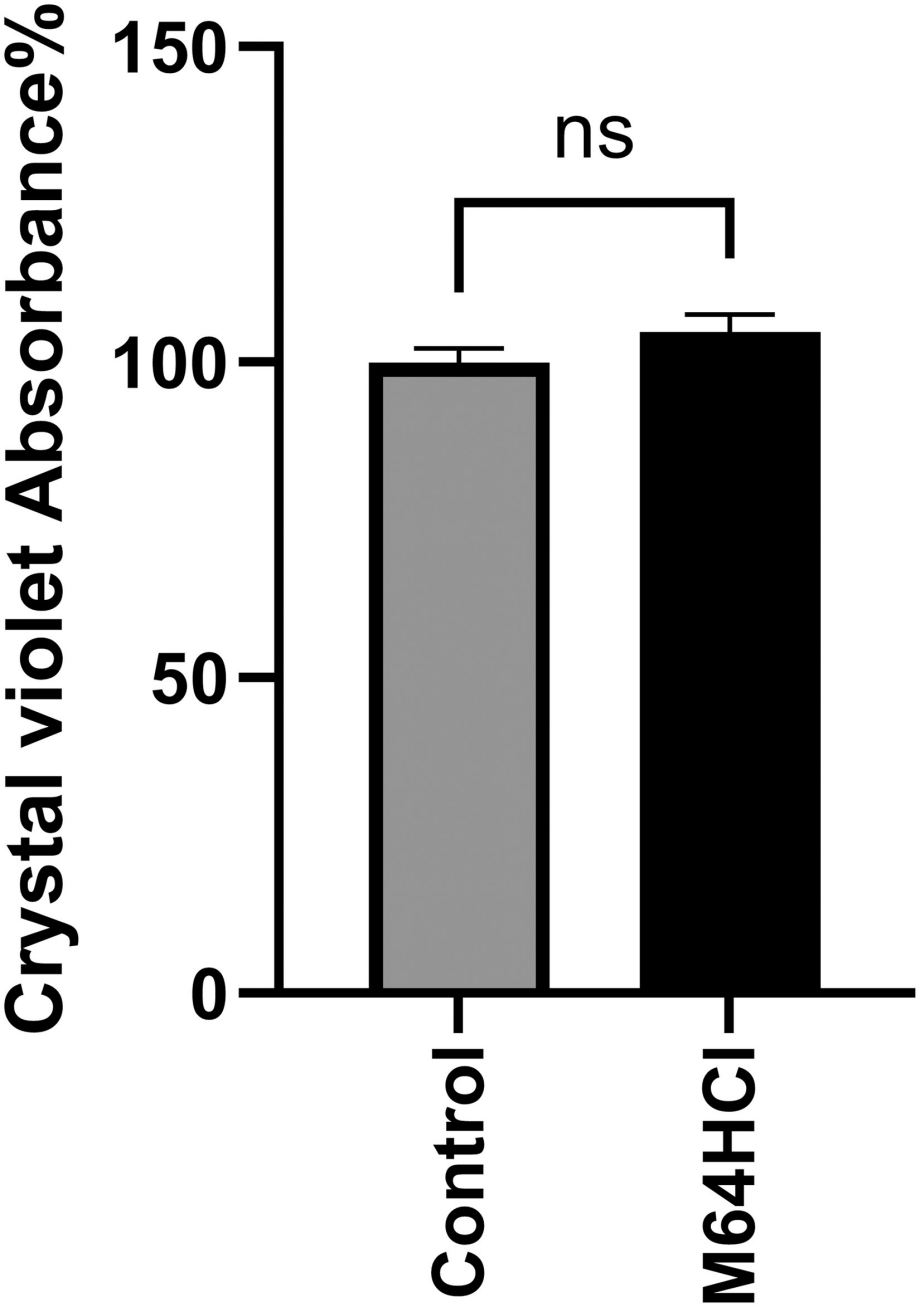
No change was observed in Caco-2 cell number, as measured by crystal violet absorbance, after treatment with 100nM M64HCl for 24 hours in a standard cell culture medium. (n = 16, pooled from three separate experiments with similar results).

Although we had previously reported that M64HCl can stimulate Caco-2 monolayer wound closure even if proliferation is blocked [11], we directly measured cell numbers after 24-hour treatment with 100nM M64HCl as an additional control. Compared to the addition of an equivalent volume of water as a vehicle control, adding M64HCl to the complete cell culture medium at a final concentration of 100nM for 24 hours did not increase cell number (Fig 3).

**Fig 4:**
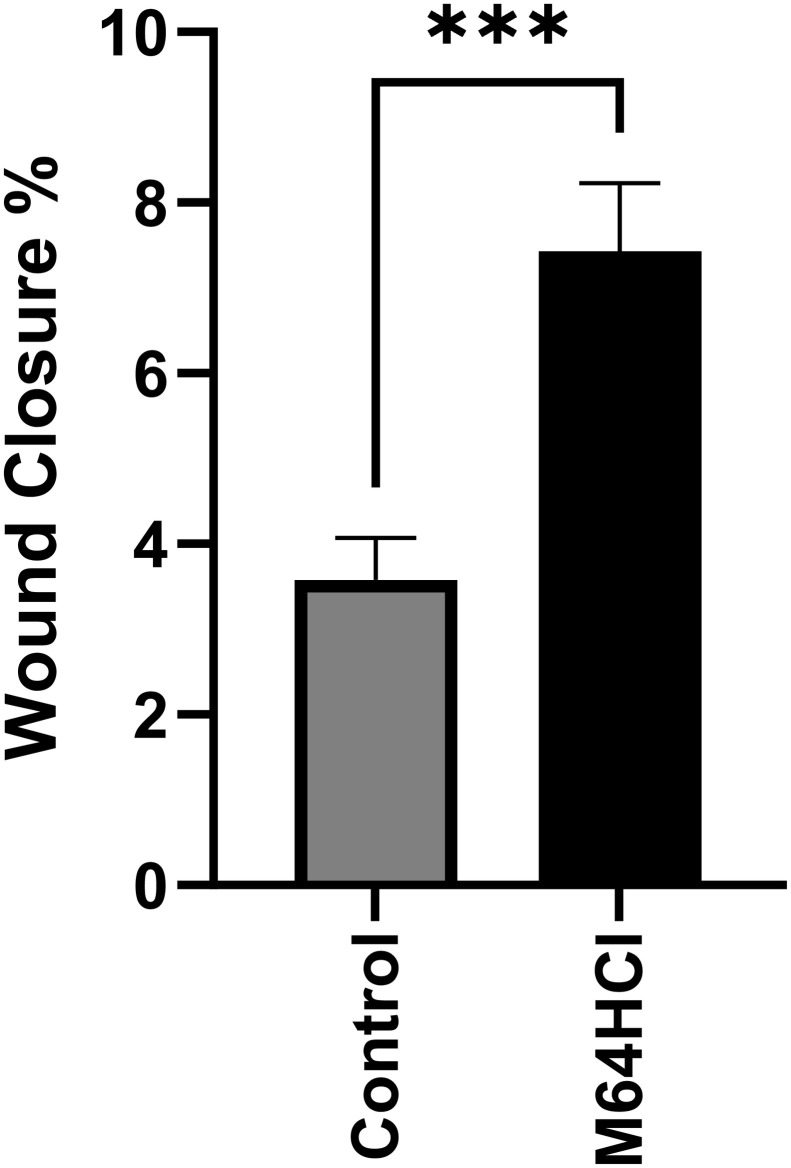
Caco-2 monolayer wound closure is accelerated by 100nM M64HCl in cell culture medium without serum. (n = 24–31, pooled from three separate studies with similar results, ***p<0.001).

It might also be important to understand whether the effects of M64HCl require joint signaling by growth factors and cytokines that are present in the fetal bovine serum of the cell culture medium and the extracellular milieu in vivo. To address this question, we performed additional experiments in which the cells were initially cultured to confluence in standard cell culture medium containing 10% FBS, then washed three times with PBS and then cultured in serum-free but otherwise identical cell culture medium prior to wounding and treatment with vehicle control or 100nM M64HCl. As expected, basal rates of wound closure without M64HCl were markedly slower in the absence of serum, but M64HCl nevertheless accelerated wound closure (Fig 4).

**Fig 5:**
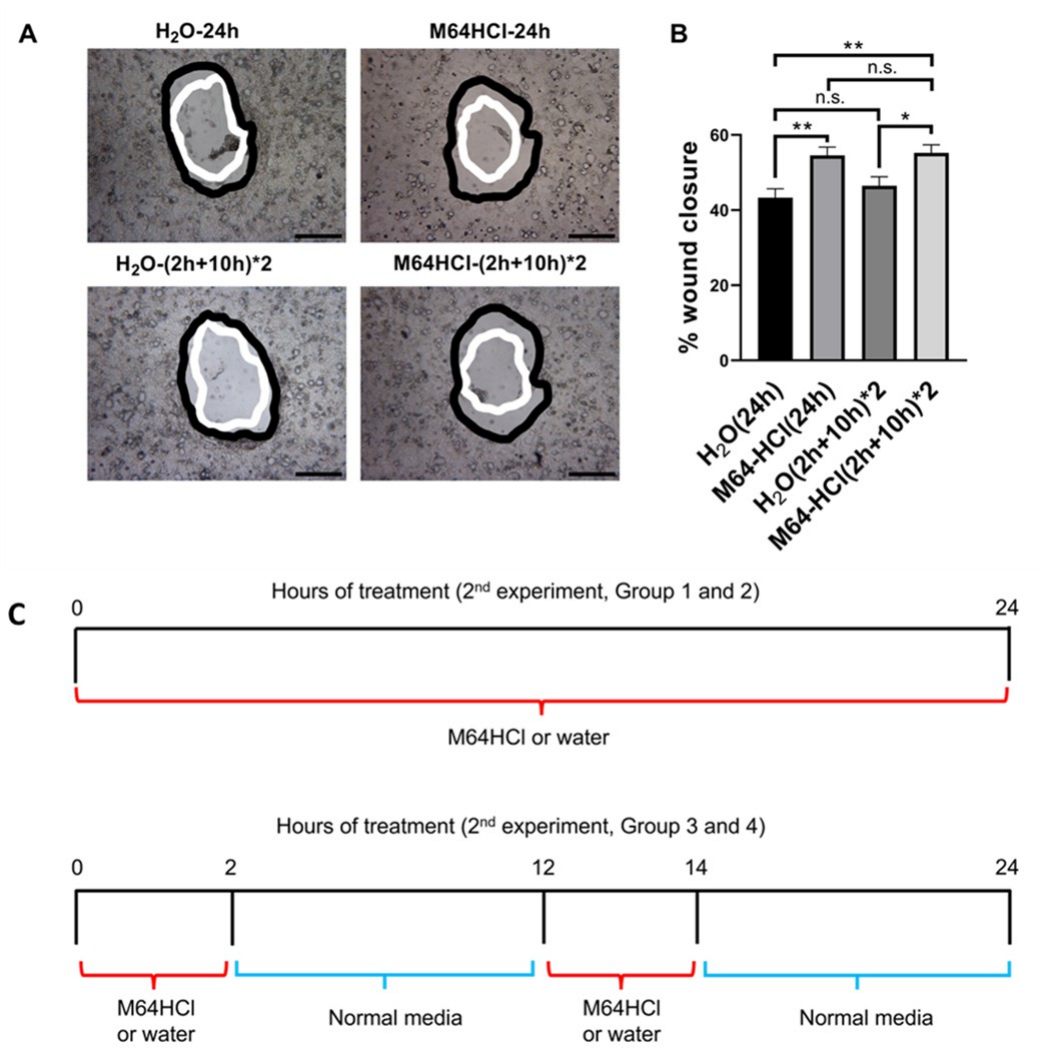
Exposing cells twice with 2-hr M64HCl treatment during a 24-hour period stimulated prolonged epithelial monolayer wound closure in Caco-2 cells. (A) Typical wound images for Caco-2 cells treated with H_2_O and M64HCl (100 nM) for 24 hours and two times two hours at 0- and 24-hour time points. All images are 4x magnification. Scale bar: 500 μm. (B) M64HCl at 100 nM accelerates circular wound closure in Caco-2 cell monolayers on collagen with both 24-hour and two times two-hour treatments (n = 20, pooled from 5 separate studies with similar results, *p < 0.05, **p < 0.01. n.s.: not significant). Black represents the circumference of the 0-hour wounds; white represents the circumference of the wounds 24 hours later. (C) Monolayer wound closure experimental design.

After we demonstrated that a single dose of M64HCl promotes prolonged monolayer wound closure with as little as 3 hours of treatment, we next sought to evaluate the effects of repeated M64HCl treatment of shorter duration on monolayer epithelial wound closure. In comparison to H_2_O treatment for 2 hours twice within 24 hours, 100nM M64HCl for 2 hours twice within 24 hours increased monolayer wound closure in Caco-2 cells (n = 20, p < 0.05, Fig 5A and 5B). Compared with the H_2_O (24h) vehicle control, incubation with 100nM M64HCl for 2 hours twice within 24 hours stimulated monolayer wound closure in Caco-2 cell by 27.4 ± 4.9% (n = 20, p < 0.01, Fig 5A and 5B) similar to the 25.9 ± 4.9% wound closure achieved by continuous treatment with 100 nM M64HCl (n = 20, p < 0.01).

### FAK inhibitor and ERK inhibitor prevent M64HCl stimulation of monolayer wound closure

In order to address the potential roles of FAK, ERK, and ROCK in possibly mediating the effects of M64HCl on wound closure, Caco-2 cells were seeded at 80% confluence into 6 well plates pre-coated with type-I collagen. When the cells reached 100% confluence, the monolayers were wounded with 200 μl non-barrier autoclaved pipette tips. Images were captured at the 0-hour time point. Then, the wounds were treated with 100 nM M64HCl,10 μM FAK inhibitor PF-573228, combined PF-573228 + M64HCl at the same concentrations, 20 μM ERK inhibitor PD 98059, combined PD-98059+ M64HCl at the same concentrations, or equivalent vehicle controls. Images were taken after 24 hours of incubation, and the wound areas were measured with Image J software.

**Fig 6:**
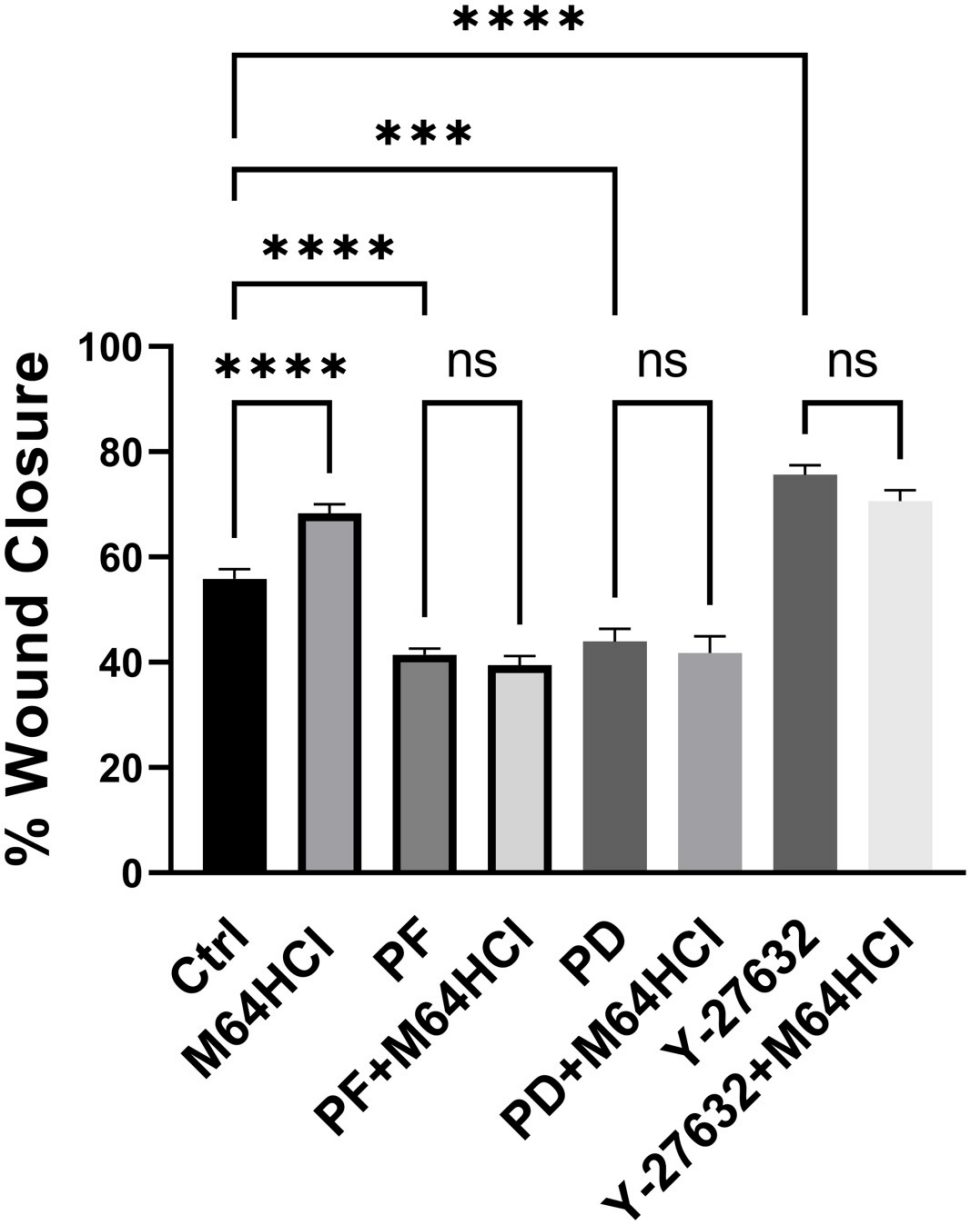
Effect of FAK inhibitor, ERK inhibitor, and Rho kinase inhibitor on basal M64HCl-stimulated monolayer wound closure. PF represents the FAK inhibitor PF-573228 (10 μM), PD represents the ERK inhibitor PD 98059(20 μM), and Y-27632 represents the Rho Kinase inhibitor Y-27632 (20 μM). (n = 12, *** p<0.0002, **** p<0.0001, n.s.: not significant).

The FAK inhibitor PF-573228 (10 μM) reduced basal monolayer wound closure by 14.4+/- 2.6%. (Fig 6, n = 12, p<0.0001) and completely prevented any increase in wound closure by M64HCl. The ERK inhibitor PD 98059(20 μM) reduced basal monolayer wound closure by 11.9+/- 2.7% and also completely prevented any increase in wound closure by M64HCl. (Fig 6, n = 12, p<0.0002). Most pertinently, either inhibition of FAK or ERK completely prevented any stimulation of wound closure by M64HCl.

### Rho kinase inhibitor increases monolayer wound closure

Caco-2 cells were seeded at 80% confluence into 6 well plates pre-coated with type-I collage. When the cells reached 100% confluence, they were wounded with 200 μl non-barrier autoclaved pipette tips. Images were captured at 0hr time point, then the wounds were treated with vehicle controls, M64HCl (100 nM), Rho Kinase inhibitor Y-27632 (20 μM), or the combination of Y-27632+ M64HCl at the same concentrations respectively. Images were taken after 24 hours of incubation, and the wound areas were measured with Image J software. Y-27632 (20 μM) stimulated Caco-2 monolayer wound closure from 55.87±2.6% to 75.63±2.7% of the original wound area (Fig 6 n = 12, p <0.0001). Simultaneous treatment with Y-27632 and M64HCl did not stimulate wound closure more than treatment with Y-27632 alone.

### M64HCl increases Rho kinase activity

**Fig 7:**
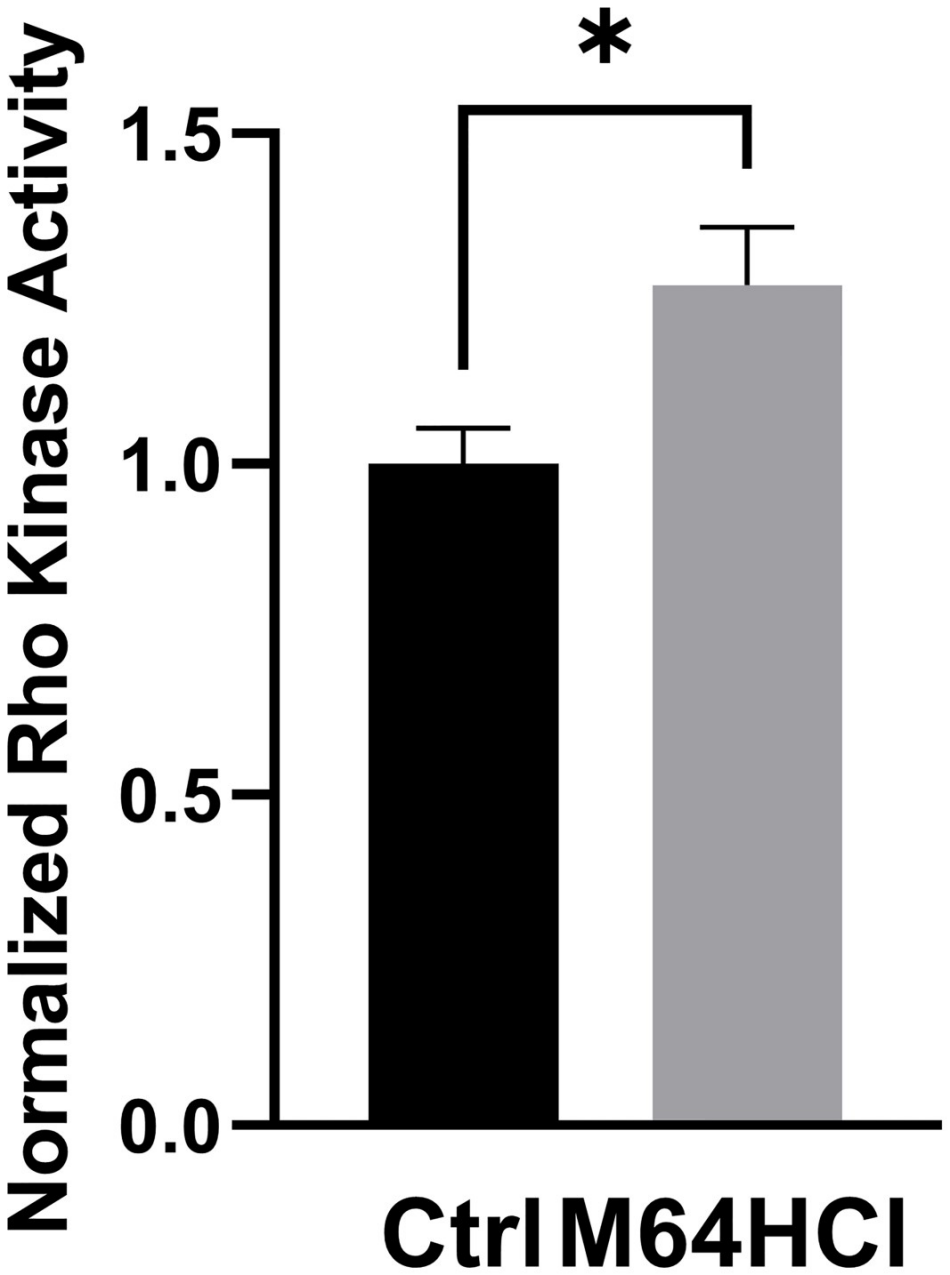
Effect of M64HCl on Rho kinase activity. (n = 12, *p<0.05).

Compared with the water vehicle control, incubation with 100nM M64HCl for 3 hours increased Rho kinase activity in Caco-2 cells by 27.1±5.6% (Fig 7 n = 12, p <0.05).

### M64HCl treatment decreases F-actin in Caco-2 cells

**Fig 8:**
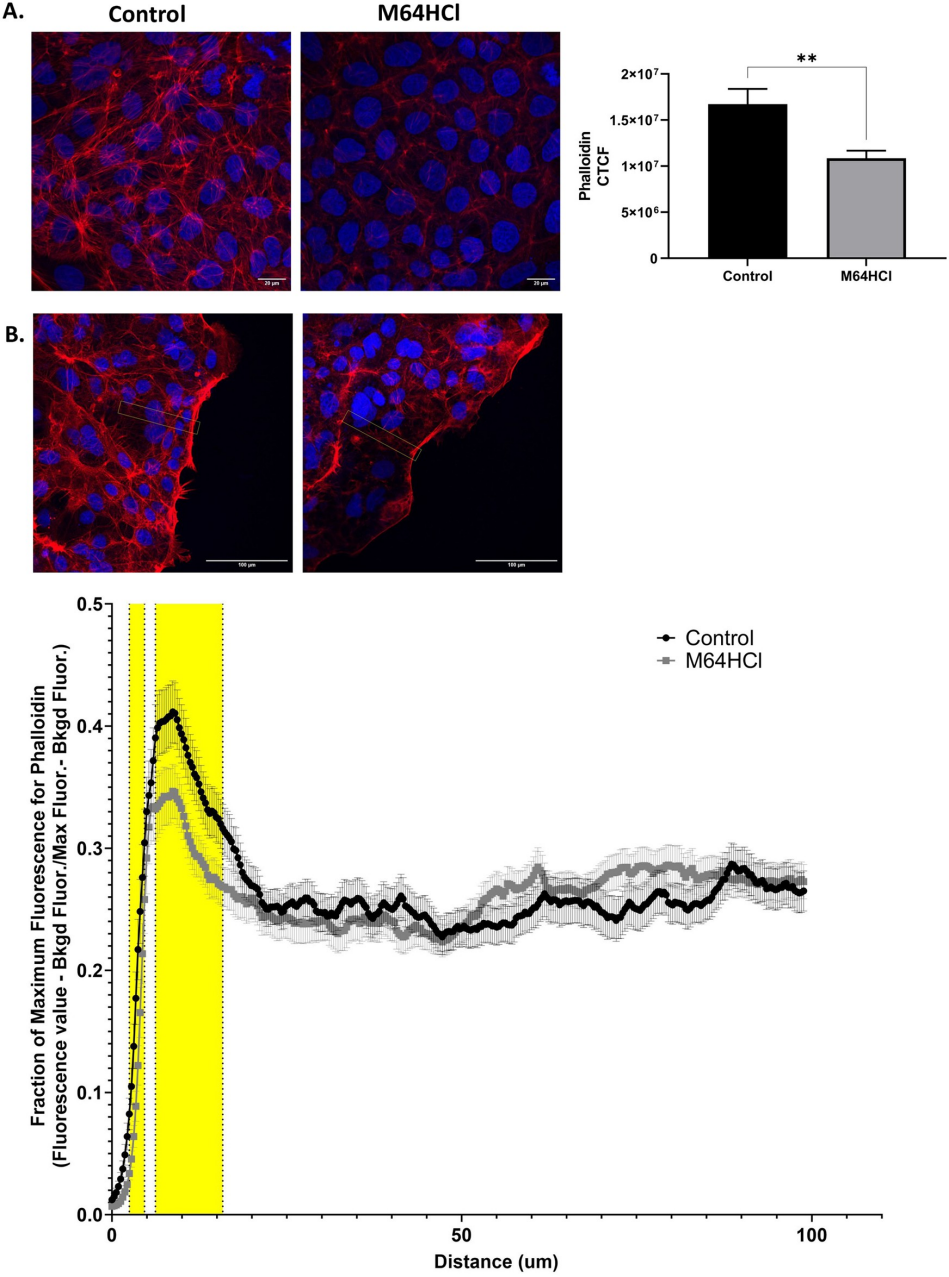
F-actin decreases after M64HCl treatment in Caco-2 cells. (A) Uninjured or (B) scratch-wounded Caco-2 cells were stained for F-actin with phalloidin (red) and nuclei with DAPI (blue) and imaged at 63x/1.4 oil with a Leica DMi8 Stellaris (for uninjured cells) or an Olympus FV3000 confocal microscope at 40x/1.4 oil (for scratch wounded cells). (n = 7–8 different sample slides per group with 9 replicates measured in each slide for uninjured slides and n = 6–7 different sample slides per group were measured for scratch wounded slides with 100 measurements taken over the 6–7 slides for each group. Additionally, 319 measurements are taken over the 100um rectangle.; * or yellow shaded regions indicate p < 0.05; yellow lined rectangles are representative regions where plot profile fluorescence was measured)”.

Caco-2 cells that were incubated with 100nM M64HCl for 24 hours and had a decrease in phalloidin fluorescence indicating a decrease in F-actin (Fig 8A). Additionally, we observed a significant decrease in F-actin phalloidin-fluorescent labeling after scratch wounding near the migrating edge (significance is yellow shaded regions from 2.486–4.661 um and 6.215–15.848 um from the migrating edge to the migrating sheet in Fig 8B). We also attempted to measure F-actin in the entire treated cell population (as opposed to only the cells in the migrating front) with fluorescently labeled phalloidin by flow cytometry but found no significant difference (S1 Fig).

### M64HCl stimulated sustained FAK (pFAK-Y-397) activation in migrating Caco-2 cells

**Fig 9:**
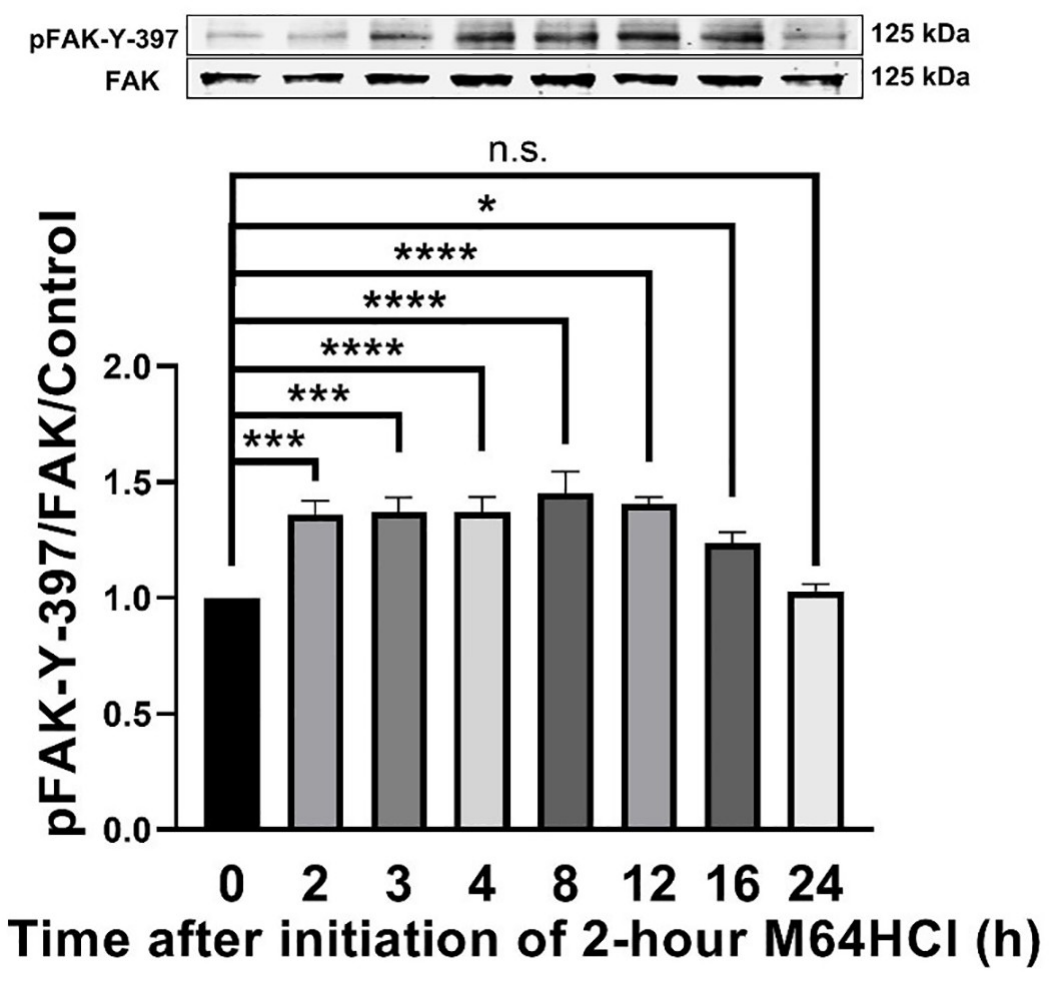
Two-hour M64HCl treatment stimulated sustained FAK activation for up to 16 hours after the initialization of the two-hour M64HCl treatment in Caco-2 cells. Caco-2 cells were treated with water as vehicle control or with 2-hour 100 nM M64HCl and harvested at indicated time points after the initiation of two-hour M64HCl treatment. Total FAK served as a loading control and internal reference. Representative blots and FAK-Y-397/FAK fold change in Caco-2 cells. (n = 6–10, *p < 0.05, ***p < 0.001, ****p < 0.0001, n.s.: not significant).

Having established that M64HCl promoted prolonged acceleration of monolayer wound closure in Caco-2 cells, we next turned our attention to intracellular signaling after M64HCl treatment. Two-hour M64HCl (100 nM) treatment increased FAK-Y397 phosphorylation over 2 to 8 hours after initiation of the 2-hour treatment with a maximum effect of 58.1 ± 7.9% (n = 10, p < 0.0001) at eight hours after the initiation of the two-hour M64HCl treatment in comparison to H_2_O vehicle control (Fig 9, n = 10). Although FAK phosphorylation began to decrease 12 hours after the initiation of the two-hour M64HCl treatment, it remained statistically higher than baseline until sixteen hours after the initiation of the two-hour M64HCl treatment, while FAK activation returned to baseline by 24 hours (Fig 9). These results demonstrated that the two-hour M64HCl treatment continued to increase FAK activation for up to 16 hours after the initialization of the two-hour M64HCl treatment or up to 14 hours after the molecule had been removed from the medium.

### M64HCl triggered sustained ERK1/2 activation (pERK1/2 T-202/Y-204) in migrating Caco-2 cells

**Fig 10:**
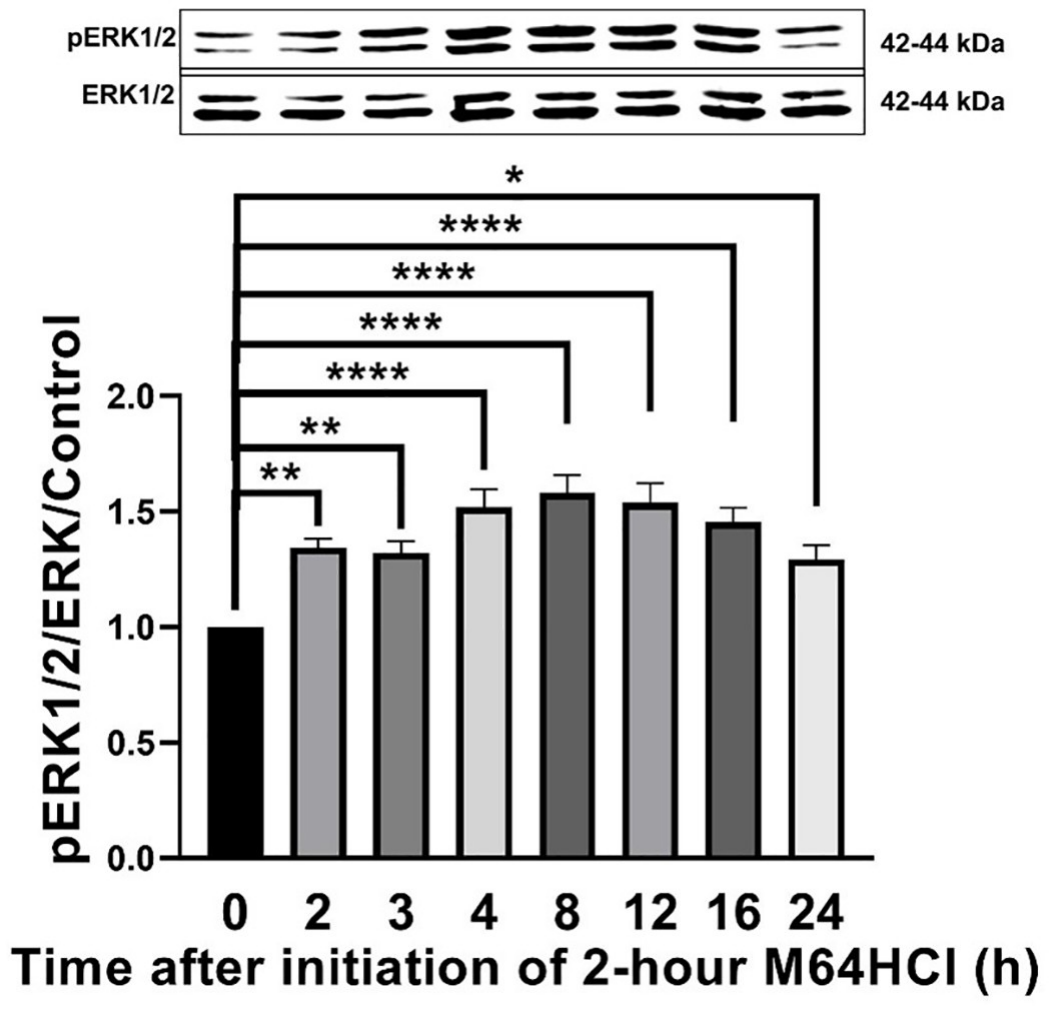
Two-hour M64HCl treatment triggered sustained ERK1/2 activation for up to 24 hours after the initialization of the two-hour M64HCl treatment in Caco-2 cells. Caco-2 cells were treated with water as vehicle control or with 2-hour 100 nM M64HCl and harvested at indicated time points after the initiation of two-hour M64HCl treatment. Total ERK1/2 served as a loading control and internal reference. Representative blots and pERK1/2 T-202/Y-204/ERK1/2 fold change in Caco-2 cells. (n = 6–10, *p < 0.05, **p < 0.01, ****p < 0.0001).

Two-hour M64HCl (100 nM) treatment increased ERK 1/2 T-202/Y-204 phosphorylation over 2- to 8 hours after the initiation of the 2-hour treatment with a maximum effect of 62.1 ± 8.2% (n = 10, p < 0.0001) (n = 10, p < 0.05) eight hours after the initiation of the two-hour M64HCl treatment in comparison to H_2_O vehicle control (n = 10) (Fig 10). Although ERK1/2 activation began to decrease twelve hours after the initiation of the two-hour M64HCl treatment, it remained substantially and statistically higher than baseline for the entire duration of the experiment until 24 hours (Fig 10). These results demonstrated that the two-hour M64HCl treatment continued to increase ERK1/2 activation for up to 24 hours after the initiation of the two-hour M64HCl treatment, or up to 22 hours after the M64HCl had been removed.

### The effect of M64HCl, FAK inhibitor PF573228, and ERK kinase (MEK) inhibitor PD98059 on phosphorylation of FAK after 24-hour treatment in migrating Caco-2 cells

**Fig 11:**
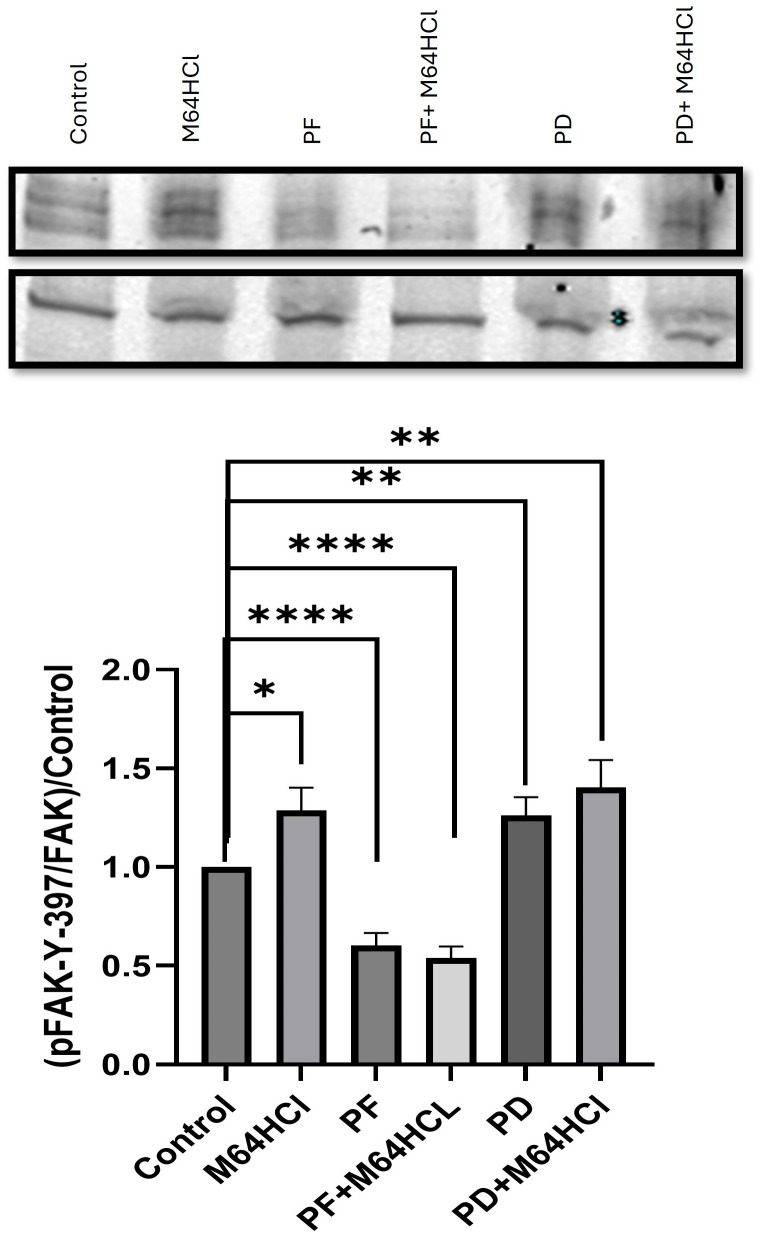
24-hr treatment with 100nM M64HCl, 10μM PF573228, the combination of 100nM M64HCl and 10μM PF573228, 10μM PD98059, or the combination of 100nM M64HCl and 10μM PD98059 in migrating Caco-2 cells. Total FAK served as loading control and internal reference. (n = 16–21, *p < 0.05, **p < 0.01, ****p < 0.0001). Representative blots for pFAK and FAK at 125 kDa are shown.

Phosphorylation of FAK in migrating Caco-2 cells significantly increased with 24-hour treatment with 100nM M64HCl by 1.29+0.11 fold (Fig 11). Treatment with 10μM of PD98059 for 24 hours resulted in a 1.26+0.09 fold increase in FAK activation while treatment with both 100nM M64HCl and 10μM of PD98059 together resulted in a 1.4+0.14 fold increase (Fig 11). However, treatment with either 10μM of PF573228 or the combination of 100nM M64HCl and 10μM of PF573228 each reduced FAK phosphorylation to 0.6+0.06 or 0.54+0.06 fold respectively (Fig 11). (P<0.05, n≥16 for all comparisons).

### The effect of M64HCl, FAK inhibitor PF573228, and ERK kinase (MEK) inhibitor PD98059 on phosphorylation of ERK1/2 after 24-hour treatment in migrating Caco-2 cells

**Fig 12:**
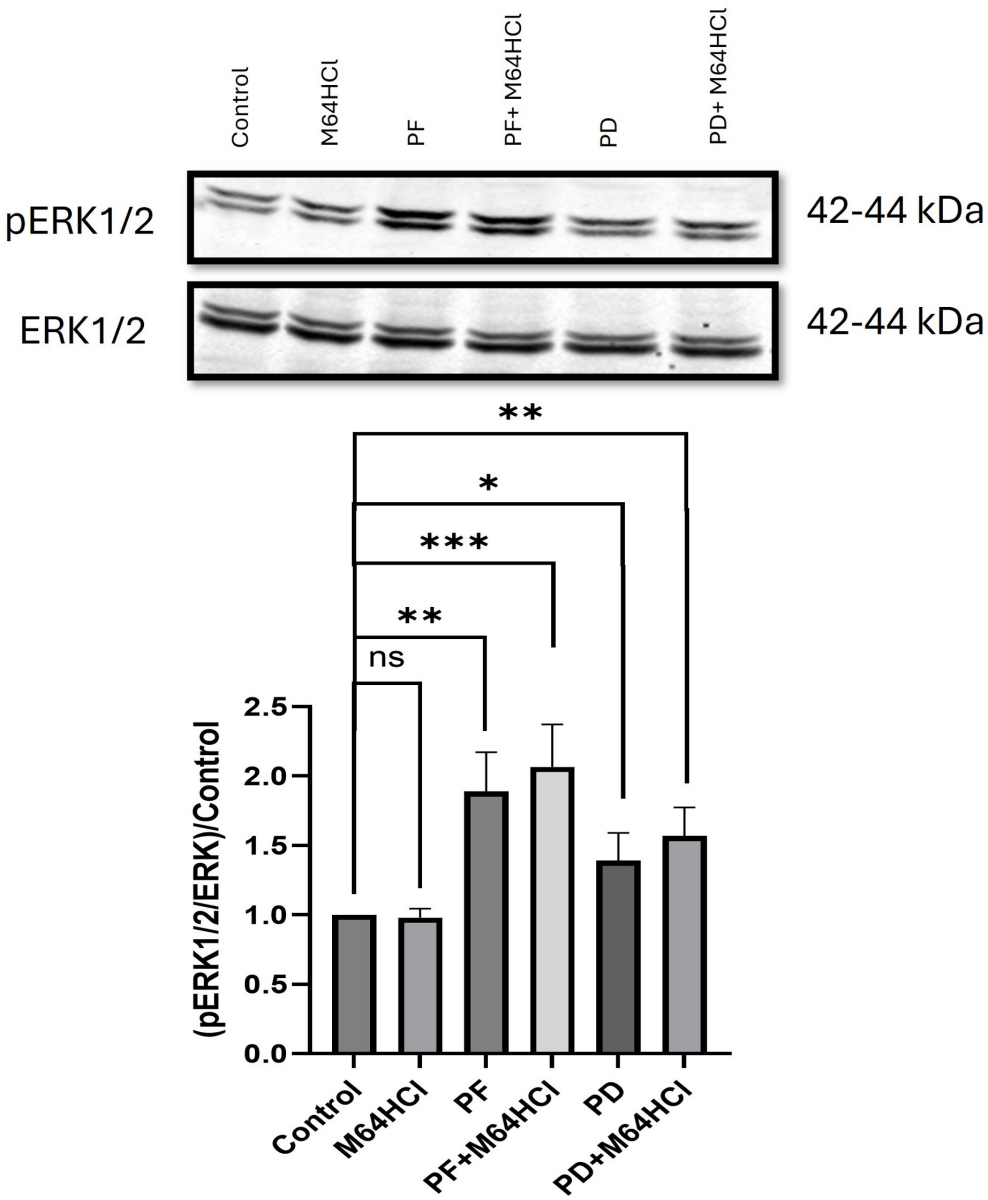
24-hr treatment with 100nM M64HCl, 10μM PF573228, the combination of 100nM M64HCl and 10μM PF573228, 10μM PD98059, or the combination of 100nM M64HCl and 10μM PD98059 in migrating Caco-2 cells. Total ERK served as loading control and internal reference. (n = 17–19, ns = not significant, *p < 0.05, **p < 0.01, ***p < 0.001). Representative blots for pERK1/2 and ERK at 42–44 kDa are shown.

Treatment with either 10μM PF573228 or the combination of 10μM PF573228 and 100nM M64HCl increased ERK 1/2 phosphorylation by 1.89+0.28 or 2.07+0.31 fold respectively after 24-hr treatment (Fig 12). Treatment with M64HCl alone did not result in a significant difference in phosphorylation of ERK1/2 after the 24-hr treatment, but interestingly treatment with either 10μM PD98059 or the combination of 10μM PD98059 and 100nM M64HCl elevated ERK1/2 phosphorylation by 1.39+0.2 or 1.57+0.2 fold respectively (Fig 12). (P<0.05, n≥16 for all comparisons).

### The effect of 1-hr treatment of PF573228 and PD98059 on ERK1/2 activation in Caco-2 cells

**Fig 13:**
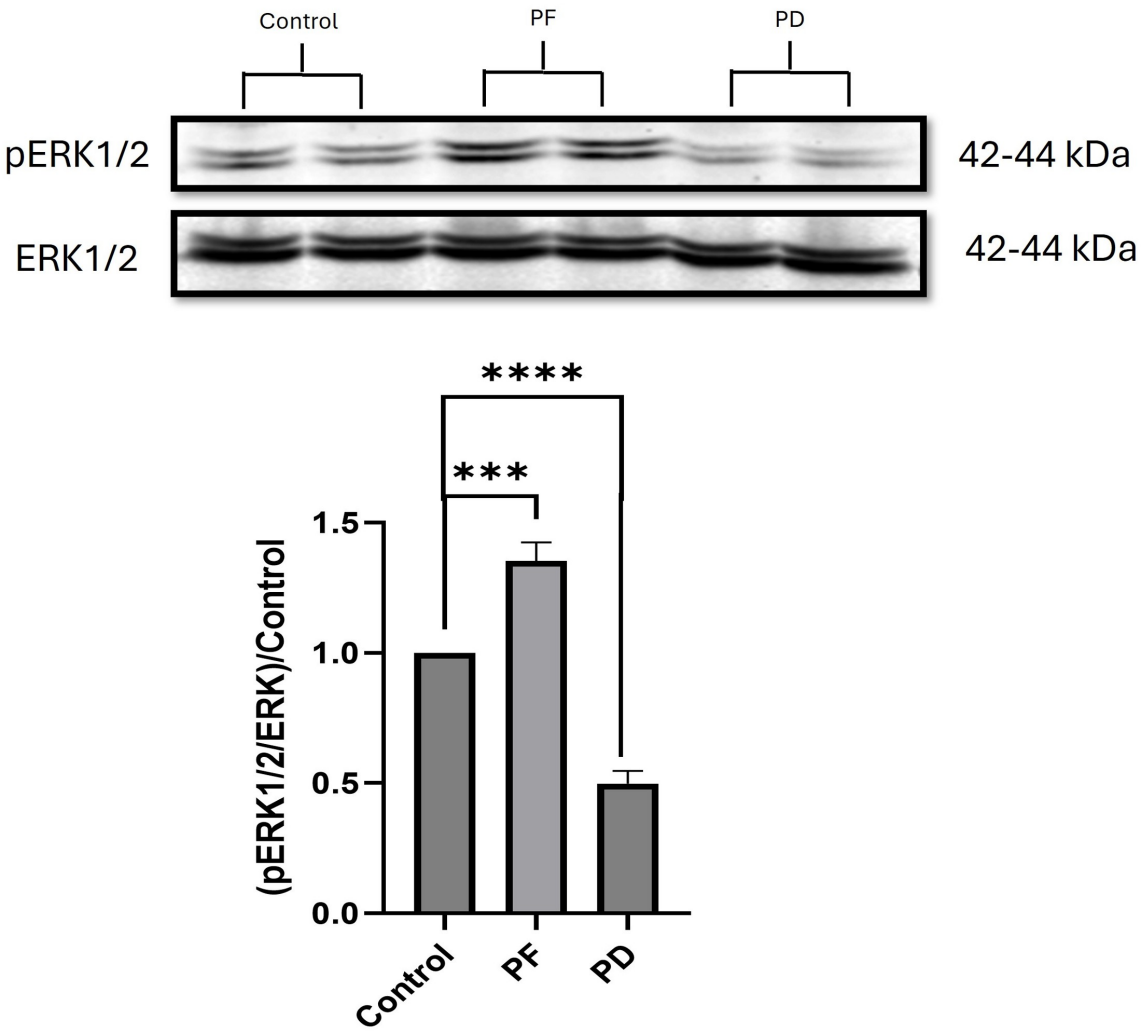
1-hr treatment with 10μM PF573228 or 10μM PD98059 in Caco-2 cells. Total ERK served as loading control and internal reference. (n = 10, ***p < 0.001, ****p < 0.0001). Representative blots for pERK1/2 and ERK at 42–44 kDa are shown.

Because the effects of PD98059 seemed to wane by 24 hours while treatment with the FAK inhibitor PF573228 resulted in ERK activation despite FAK inhibition, we then conducted an additional series of experiments confirming the effects of PF573228 and PD98059 on ERK activation after one-hour treatment. We treated Caco-2 cells with 10μM PF573228 or 10μM PD98059 for one hour. Treatment with the FAK inhibitor 10μM PF573228 resulted in 1.4+0.07 fold increased ERK1/2 phosphorylation (Fig 13). (n = 10, p<0.001). In contrast, one-hour treatment with the MEK1 inhibitor 10μM PD98059 substantially reduced ERK1/2 phosphorylation 0.5+0.05 fold (Fig 13). (n = 10, p<0.0001).

## Discussion

Traditional therapies for gastrointestinal mucosal lesions aim to reduce or neutralize the noxious factors that injure the gut with medications such as proton pump inhibitors (PPIs), histamine-2 receptor antagonists (H2-antagonists), antibiotics, corticosteroids, and immunosuppressants. These therapeutics contribute to gastrointestinal mucosal healing by inhibiting the underlying pathology without directly promoting mucosal restitution. Indeed, some of these drugs such as PPIs and H2-antagonists, in fact, worsen lower GI injury by increasing the gastric pH [3, 4, 17]. There is, therefore, a need for a new therapeutic agent that directly promotes both upper and lower GI mucosal repair. In particular, while both cell motility and cell proliferation are important for wound healing, we sought an agent that would promote epithelial cell motility. Epithelial wound closure, whether in vitro or in vivo, represents a combination of cell motility and cell proliferation. We have previously reported that the effects of FAK activating molecules including the prototype ZN27 [7] and M64HCL itself [12] are not blocked by hydroxyurea. Indeed, although we have not done these studies in vivo for M64HCl itself, no increase in Ki-67 staining was observed in vivo during the stimulation of jejunal mucosal wound healing by the prototype ZN27 molecule [7]. Our new experimental data demonstrates no change in Caco-2 cell number after treatment with M64HCl at the concentration demonstrated here to promote monolayer wound closure. While this in no way obviates the contribution of cell proliferation to epithelial wound healing, these data strongly suggest that the effect of M64HCl studied here is motogenic rather than mitogenic.

In addition, epithelial cell migration is regulated by a complex web of signals derived from soluble growth factors (including those in the bloodstream) and matrix-bound molecules. It is therefore not surprising that we found wound closure to be slower in serum-free medium. However, it was noteworthy that FAK activation by M64HCl demonstrably accelerated wound closure even in the absence of growth factors or other motogenic signals in serum.

We previously reported that the FAK activator ZN40099027 enhances FAK activity via interaction with the FAK kinase domain to increase the Vmax of FAK for adenosine triphosphate (ATP) [9]. ZN40099027 at 10 nM activates FAK by 14.8% in suspended Caco-2 cells [7]. We recently described a library of novel FAK small-molecule activators that activate FAK and accelerate in vitro wound closure at concentrations as low as 100 pM while promoting small bowel mucosal wound healing in mice over three days and without obvious toxicity [11]. However, these FAK activators, like ZINC40099027 and other molecules so far studied, all required DMSO solubilization with attendant DMSO toxicity.

M64HCl is more potent in that it activates FAK at concentrations as low as 100 pM, and produces a 20.2% activation at 10 nM. M64HCl is a water-soluble small molecule that has drug-like physicochemical properties. Its molecular weight <500, solubility> 100 μM, and log D between 0 and 3 all enhance the probability of good intestinal permeability. Indeed, it is rapidly absorbed after enteral gavage and highly concentrated in the gastric and small intestinal mucosa. M64HCl promotes gut mucosal healing in mice after injury with minimal toxicity [12]. However, a plasma half-life of fewer than two hours is required in vivo studies with M64HCl to necessitate continuous infusion for proof of principle [12]. In addition, all of our previous studies of FAK-activating molecules have involved 24-hour treatment. However, it is not uncommon for a drug to have effects that extend beyond its half-life [18, 19]. Therefore, it seemed desirable to investigate the duration of FAK activation and epithelial sheet migration after M64HCl treatment to clarify whether a shorter exposure to a small molecule FAK activator might have sustained effects. The present study importantly advances our knowledge in this area because it demonstrates that relatively shorter exposure to effective levels of M64HCl can have sustained effects even when the M64HCl itself has been cleared from the extracellular space. This opens the door to potential clinical translation for such molecules despite shorter half-lives. We previously reported that, in addition to conventional FAK activation in the membrane, the FAK activator ZN27 binds to the 35 kDa kinase domain of FAK and facilitates FAK Tyr-397 autophosphorylation in the cytosol before the Tyr-397 phosphorylated FAK translocates to the membrane, where it is further phosphorylated and in turn activates downstream substrates such as ERK1/2, thus stimulating human intestinal epithelial motility in vitro [10] and murine gastric [8] and small intestinal [7] mucosal healing in vivo. M64HCl similarly activates FAK dose-dependently by interacting with the FAK kinase domain [12]. It is becoming increasingly obvious that cellular signals are not only simple binary on-off switches. The type and duration of the stimulus and the cell type involved determine the duration and/or magnitude of cellular signals, and thus the effect on the cell. For instance, it is not uncommon for effects to last beyond the application of a stimulus, whether that stimulus is an endogenous agent or a drug. Thus, ibuprofen and acetaminophen, the most commonly used analgesics, have half-lives of approximately 2 hours but durations of action that extend 4–6 hours [20, 21]. Transient progesterone treatment of oocytes results in a sustained cellular response by arresting them in their mature, germinal vesicle breakdown (GVBD) state for up to several days after progesterone removal [22]. Indeed, in some other cases, stimulation of the same signaling pathway with different stimuli may affect the cellular outcome by altering the duration of the signal. For example, constant applications of epidermal growth factor (EGF) or nerve growth factor lead to transient or sustained ERK activation stimulating proliferation or differentiation, respectively in pheochromocytoma PC12 cells [23]. Moreover, the timing of the stimulus (constant vs pulsed stimulation) may also affect the cellular signals. In contrast to constant EGF stimulation, repeated pulses of 3 min EGF lead to sustained ERK activation in pheochromocytoma PC12 cells [23, 24].

Our analysis of intracellular M64HCl concentrations suggested that the two-hour M64HCl (100nM) treatment increased the intracellular M64HCl concentration approximately three-fold over the extracellular concentration, suggesting that M64HCl may be concentrated intracellularly. Our results do not rule out an active concentration mechanism but may simply be explained by M64HCl binding to FAK or other intracellular components. Some drugs may accumulate in tissues because they bind to proteins, phospholipids, or nucleic acids within cells. For instance, chloroquine concentrations in white blood cells or liver cells can reach thousands of times higher than those in plasma [25]. After the treatment, we readily washed all the M64HCl from the media, but medium concentrations increased slightly and gradually at 30 and 60 minutes after the washing steps, most likely because of the release of FAK-bound M64HCl within the cells. Conversely, washing decreased the M64HCl concentration by 95% within the cells, presumably, by removing largely unbound intracellular M64HCl rather than bound M64HCl, and we observed a corresponding slow decrease in M64HCl intracellular concentrations thereafter as the medium M6HCl increased slightly. This may be analogous to the in vivo situation where the plasma levels (and thus presumably extracellular fluid levels) decrease rapidly and dramatically due to the half-life of a drug but leaving some molecules still intracellular, either free or protein-bound, that slowly elutes out and results in a long terminal half-life for drugs having a relatively high clearance [26–28].

M64HCl treatment stimulated wound closure in Caco-2 cell monolayers regardless of the duration of M64HCl treatment or whether it was intermittent or constant. Furthermore, the motogenic effect was prolonged substantially beyond the stimulus, so that a single three-hour treatment or two two-hour treatments had equivalent effects to sustained 24-hour treatment with M64HCl. The prolonged FAK activation and even longer ERK activation after briefer exposure to M64HCl likely explain this sustained motogenic effect.

Both the FAK inhibitor and the ERK inhibitor reduced basal wound closure in the absence of M64HCl, consistent with the importance of the FAK-ERK pathway in epithelial sheet migration, and each completely prevented any stimulation of wound closure by M64HCl, demonstrating the specificity of the agent for FAK and consistent with the concept that ERK is downstream of FAK in this pathway [29–32]. FAK signaling is interconnected with various pathways including the Rho-family guanosine triphosphatase (GTP)ases RhoA, Rac, and Cdc42 [33]. Rho-kinase (ROCK) is a downstream effector of the small GTPases RhoA, B, and C [34]. FAK signaling works in cooperation with Rho-ROCK signaling to regulate cell morphology [35, 36].Global ROCK kinase inhibition by Y-27632 resulted in a stimulated effect on Caco-2 monolayer wound closure [37] and by others of similar effects that Rho-kinase inhibitor Y-27632 induces the migration of human periodontal ligament stem cells [38] and enhanced cutaneous wound closure in the majority of wounds in mice [39]. The present study demonstrated that M64HCl increases Caco-2 cell migration rate and Rho kinase activity. However, we observed that Y27632, a Rho kinase inhibitor promotes Caco-2 wound closure. The role of Rho kinase in this process is currently unclear. This needs to be investigated further in the future.

Western blotting at 24 hours confirmed increased FAK phosphorylation in response to M64HCl similar to that observed after two-hour treatment. However, we notably did not observe increased ERK1/2 activation at the 24-hour time point, unlike the effects of the shorter 2-hour treatment. This likely reflects negative feedback that counters the initial ERK activation over this longer time period, consistent with previous reports that sustained ERK activation results in a negative feedback loop [40]. For instance, activated ERK translocates into the nucleus and promotes transcription for MAP kinase phosphatases (MKPs) such as DUSP6. DUSP6 in turn inactivates ERK by dephosphorylation [40]. The FAK activation observed in response to the MEK1 blockade by PD98059 may reflect a similar counterregulatory loop, which awaits further definition.

The FAK inhibitor PF573228 blocked FAK activation as expected, consistent with its ability to reduce basal wound closure and prevent the stimulation of wound closure by M64HCl. Interestingly, treatment with PF573228 markedly activated ERK1/2 in Caco-2 cells at both one and 24 hours. This may at least in part reflect a counterregulatory feedback response to the inhibition of FAK and all of its downstream signals. For instance, reduced FAK and MAPK activation might downregulate DUSP6, resulting in consequent ERK activation [40]. While a counterregulatory loop like this would not necessarily be expected to produce an effect past the baseline, FAK inhibition is likely to have more widespread consequences than only ERK activation, and the reduction in these other signals might well further stimulate the counterregulatory loop to cause additional activation of ERK.

The combination of M64HCl with PD98059 stimulated ERK activation at 24 hours more than PD98059 alone, consistent with a model in which FAK modulation has more global and distinct consequences beyond that of its downstream effects on ERK. The blockade of M64HCl-stimulated wound closure by PD98059, therefore, may reflect either FAK-dependent but ERK-independent motogenic signaling or the consequences of ERK blockade earlier in the experiment. This interesting question awaits further exploration.

Focal adhesions play an important role in myosin II-dependent contraction, which is a main component in cellular motility in a wide variety of cell types. The cell motility process is mediated through cycles of protrusions, adhesion, and contraction through force-producing interactions of myosin II, actin, and focal adhesion points [41]. Protrusion occurs through collaborative processes between actin, capping protein, ARP2/3, linking proteins such as filamin, and polymerizing proteins such as formins [42]. Treatment with M64HCl decreased F-actin fluorescence in cells at the migrating front when compared to control in both non-injured and scratch-wounded cell cultures. The reasons for this could be the following. The area of actin concentration for treated cells when compared to control cells appeared to be smaller, condensed in areas near the cell membrane. Moreover, the contractile portion of the motility cycle could be leading to depolymerization of F actin at a greater rate. With a greater assembly of focal adhesion points, we would hypothesize that the contractile force would be greater but could also be inversely correlated to F actin fluorescence due to the shortening of the contractile unit and thus actin. Carbachol is a cholinergic agonist that is involved in promoting gastrointestinal motility, raises glandular secretion, and shields the intestinal barrier [43], and it has been shown to increase FAK Y397 phosphorylation [44, 45]. Carbachol was shown to lower the severity of intestinal injury which also correlated with a decrease in F-actin in rat intestinal epithelium with severe acute pancreatitis over the control groups [43]. Additionally, Ubelmann et al. treated mouse small intestine with carbachol and showed that the apical F-actin staining was decreased in comparison to untreated intestine [46]. Richman and Regan utilized α_2_-adrenergic receptors-selective agonist dexmedetomidine (Dex) to demonstrate an increase in ERK 1/2 kinase activity in rat aortic smooth muscle (RASM) cells, an increase in cell migration without proliferation as shown through [^3^H]thymidine incorporation, and a decrease in F-actin phalloidin labeling [47]. Sun et al. examined the migration and invasion of liver cancer stem cells after treatment with the antitumor drug, salinomycin. They found that salinomycin repressed FAK and ERK 1/2 phosphorylation, inhibited LCSC migration, while increasing cell stiffness, and was shown to be from an increase in F-actin filaments [48]. The apparent disparity between the imaging results demonstrating a decrease in F-actin and the flow cytometric results that did not identify F-actin changes after M64HCl treatment likely reflects the dilution of the effect in the cells at the migrating front by the lack of effect in cells behind the migrating front (as we observed in our imaging studies). In addition, the changes in cell geometry that accompany trypsinization and cell detachment from the substrate may also alter F-actin dynamics.

These results raise the possibility that a FAK activating molecule with a shorter half-life might nevertheless promote mucosal healing in vivo with once or twice daily dosing. The studies reported here were performed in a single cell line, the Caco-2BBE line. Although these cells are common models for intestinal epithelial non-malignant biology, there are multiple and manifest differences between the behavior of a cell line in culture and the biology of primary epithelium in vivo. Our previous demonstration that M64HCl does, indeed, heal jejunal mucosal wounds in mice [12], taken together with similar demonstrations of the efficacy of another FAK-activating molecule in various mouse models and other cell lines [7, 8] are consistent with the proposal that M64HCl or FAK-activating agents like it may promote mucosal wound closure in human tissues through a motogenic effect. However, the fundamental conclusion of this study, that shorter term or intermittent exposure to M64HCl may have equivalent motogenic effects to more constant exposure, awaits confirmation in vivo. There are obvious differences between *in vitro* and *in vivo* studies. *In vitro* experiments do not have the complexity of the organ systems or the internal environment of living organisms. *In vivo* pharmacokinetics (absorption, distribution, metabolism, and excretion) of a small molecule may alter the effects of a drug *in vivo* in ways not always obvious from cell culture studies.

M64HCl is a promising novel compound with drug-like, water-soluble properties. Since its short plasma half-life may hinder clinical use due to requirements for multiple daily dosing, it may be beneficial to modify the molecule for a longer half-life. However, this study suggests that, at least in vitro, FAK activators may not need to be continuously present in order to trigger a sustained motogenic effect, so molecular modifications that achieve a half-life of only a few hours might suffice for twice daily or even once daily dosing as an effective therapy to promote upper and lower GI mucosal healing.

## Supporting information

S1 Fig F-actin % measured by flow cytometry in the entire treated cell population. (TIF)

S1 Raw images Original images for the western blots. (PDF)
