# Supplementary figures and images for: Sustained intestinal epithelial monolayer wound closure after transient application of a FAK-activating small molecule
Source: PLoS One. 2024 Aug 16;19(8):e0304010. doi: 10.1371/journal.pone.0304010 (PMC11329154; doi:10.1371/journal.pone.0304010)

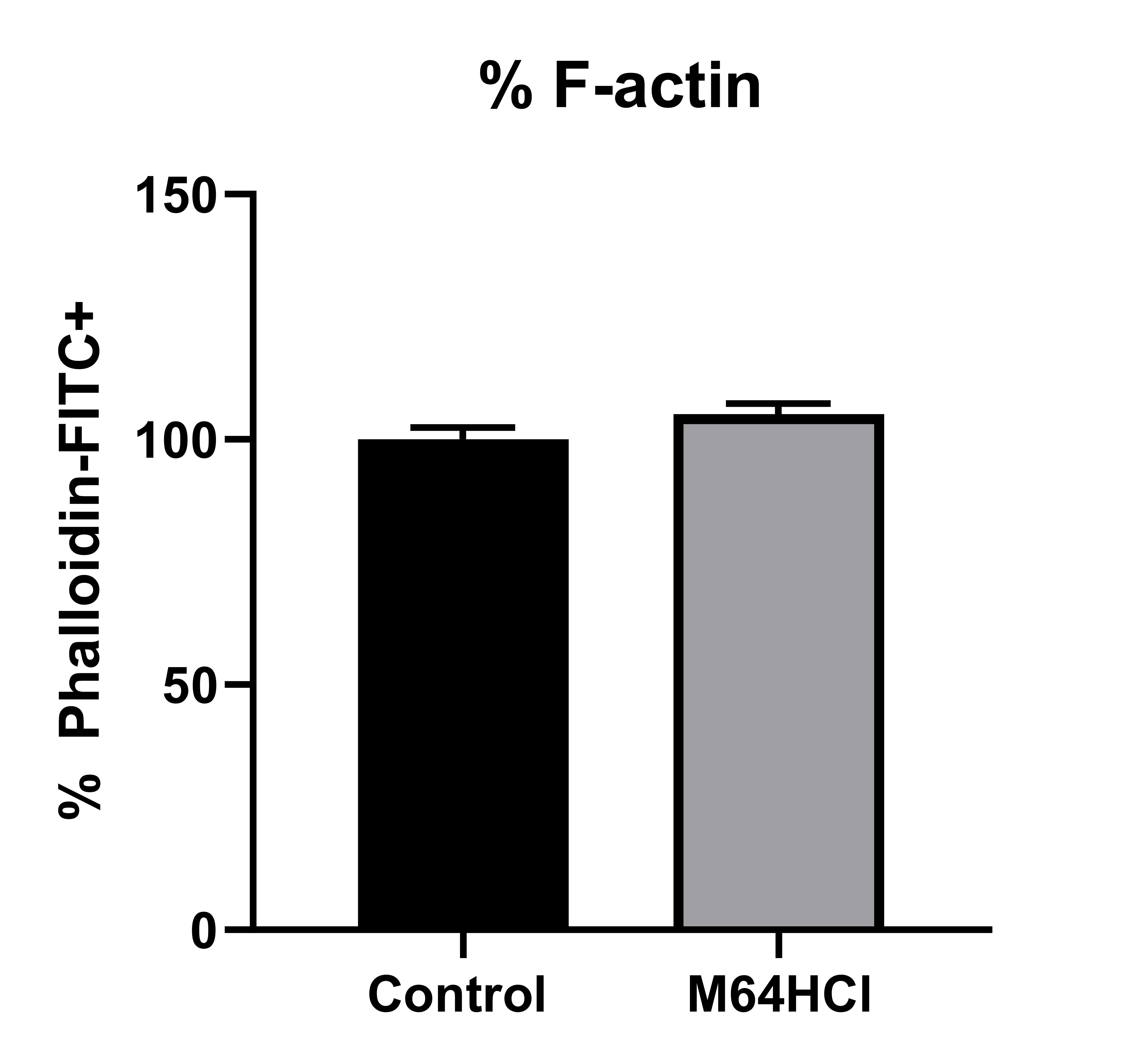

Supplement: S1 Fig — (TIF) [file pone.0304010.s001.tif]
