# Supplementary material for: Sustained intestinal epithelial monolayer wound closure after transient application of a FAK-activating small molecule
Source: PLoS One. 2024 Aug 16;19(8):e0304010. doi: 10.1371/journal.pone.0304010 (PMC11329154; doi:10.1371/journal.pone.0304010)
Supplement: S1 Raw images — (PDF) [file pone.0304010.s002.pdf]

Original Images for Blots

Fig 9. Two-hour M64HCl treatment stimulated sustained FAK activation for up to 16 hours after the initialization of the two-hour M64HCl treatment in Caco-2 cells.

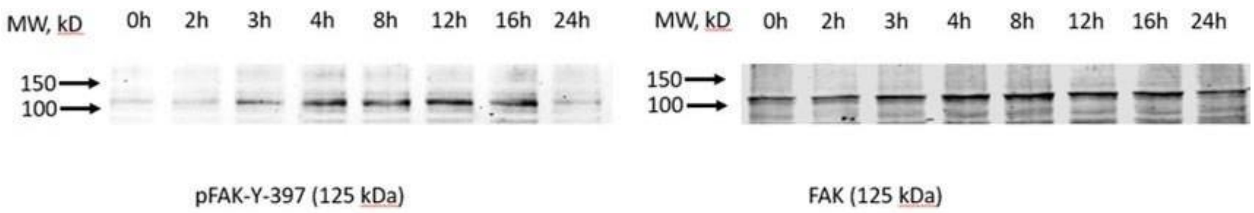

Fig 10. Two-hour M64HCl treatment triggered sustained ERK1/2 activation for up to 24 hoursafter the initialization of the two-hour M64HCl treatment in Caco-2 cells.

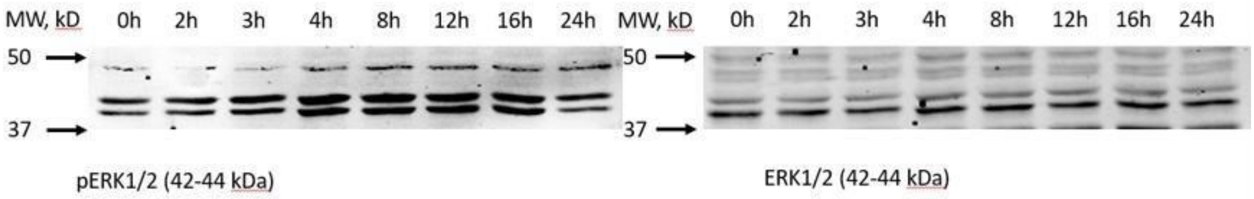

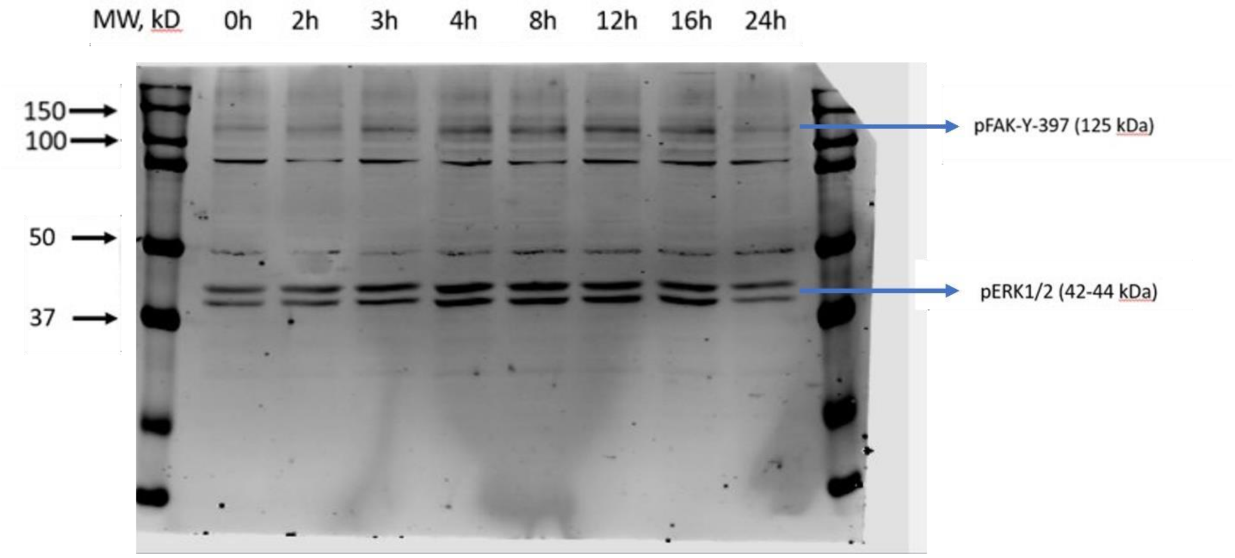

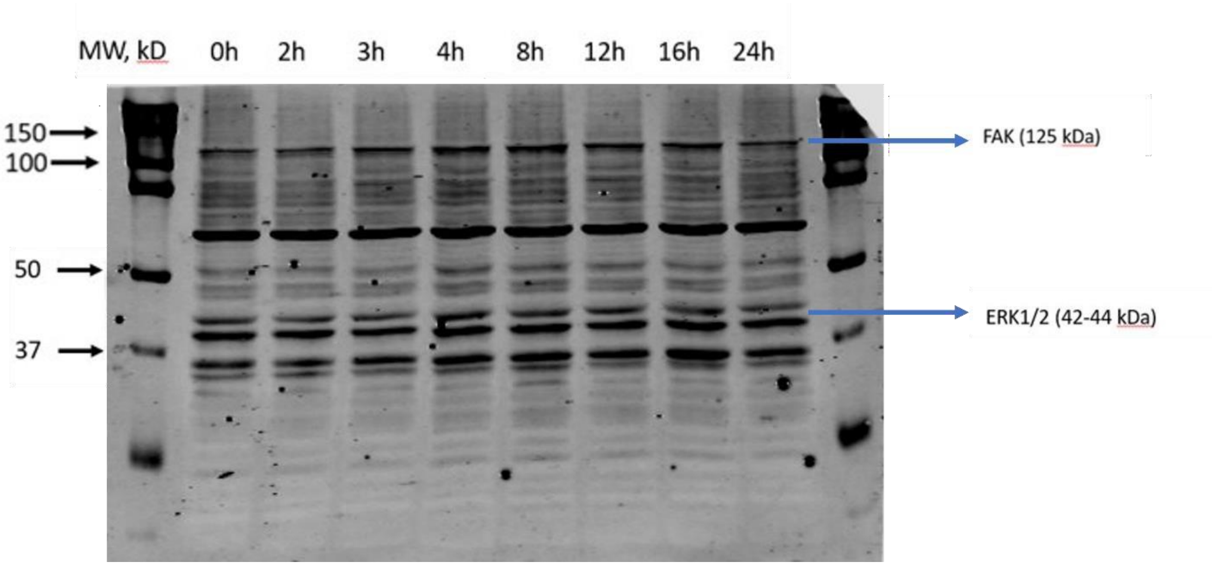

Fig 11. 24-hr treatment with 100nM M64HCl, 10µM PF573228, the combination of 100nM M64HCl and 10µM PF573228, 10µM PD98059, or the combination of 100nM M64HCl and 10µM PD98059 in migrating Caco-2 cells. Total FAK served as loading control and internal reference. (n=16-21, *$p < 0.05$, **$p < 0.01$, ****$p < 0.0001$). Representative blots for pFAK and FAK at 125 kDa are shown.

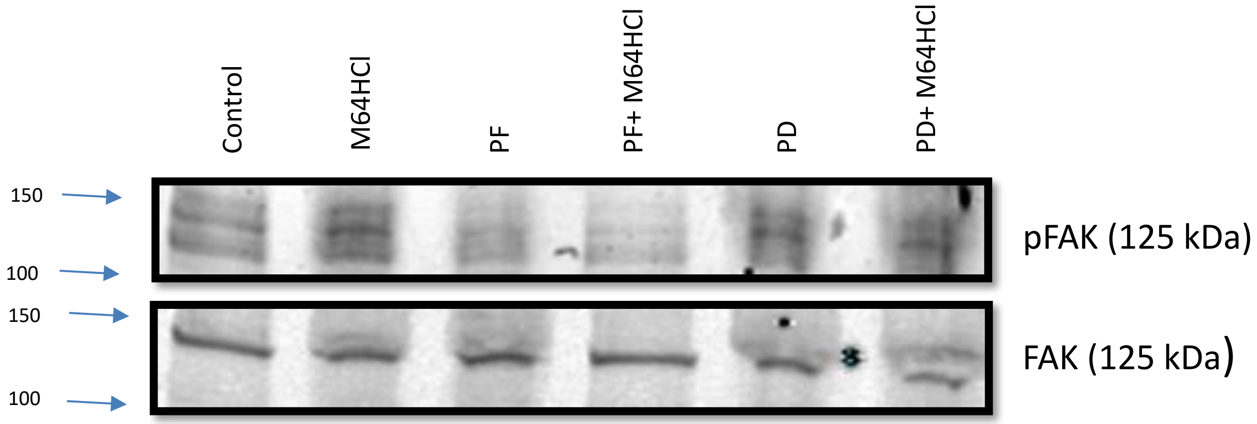

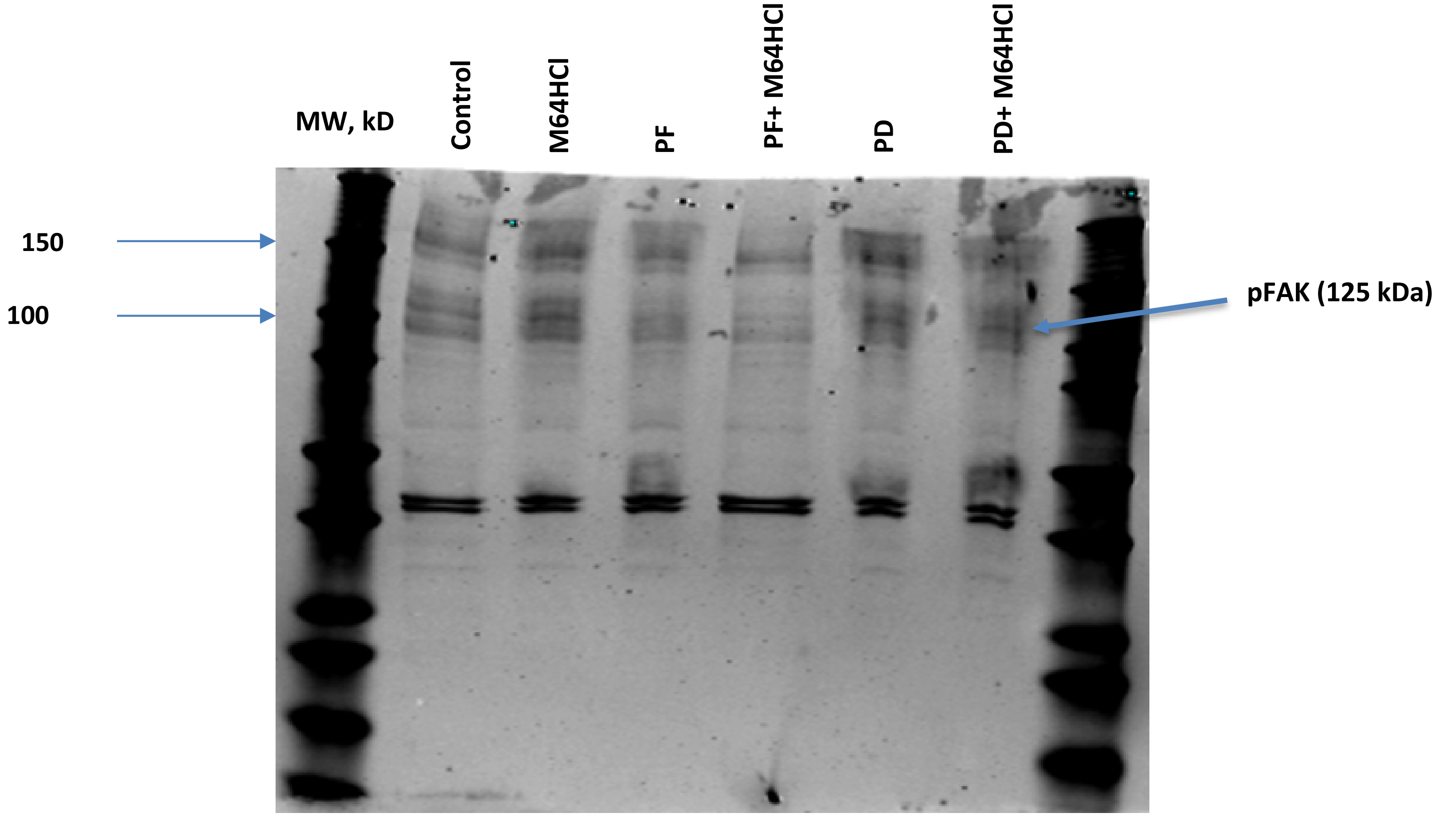
MW, kD
Control
M64HCl
PF
PF+ M64HCl
PD
PD+ M64HCl
150
100
pFAK (125 kDa)

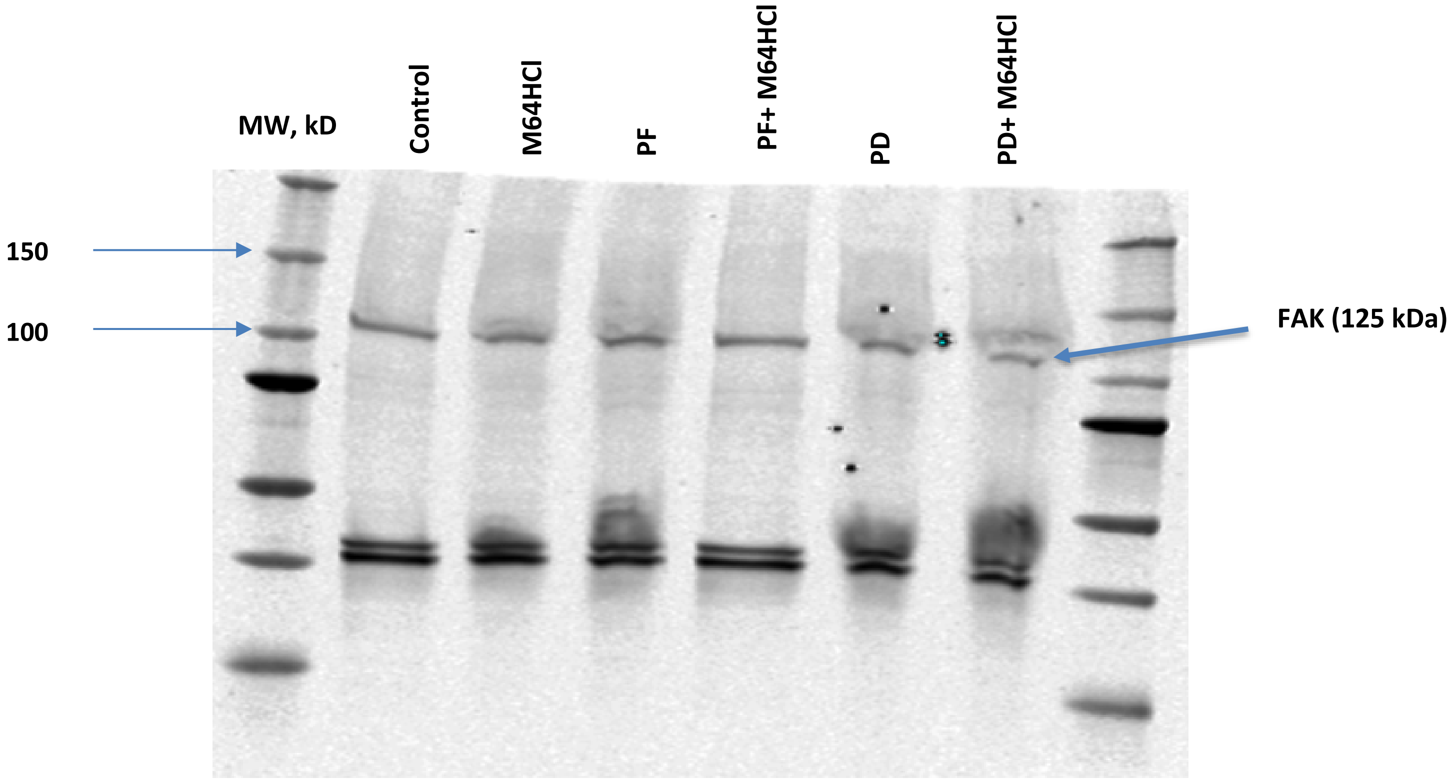
MW, kD
Control
M64HCl
PF
PF+ M64HCl
PD
PD+ M64HCl
150
100
FAK (125 kDa)

Fig 12. 24-hr treatment with 100nM M64HCl, 10μM PF573228, the combination of 100nM M64HCl and 10μM PF573228, 10μM PD98059, or the combination of 100nM M64HCl and 10μM PD98059 in migrating Caco-2 cells. Total ERK served as loading control and internal reference. (n=17-19, ns= not significant, *p < 0.05, **p < 0.01, ***p < 0.001). Representative blots for pERK1/2 and ERK at 42-44 kDa are shown.

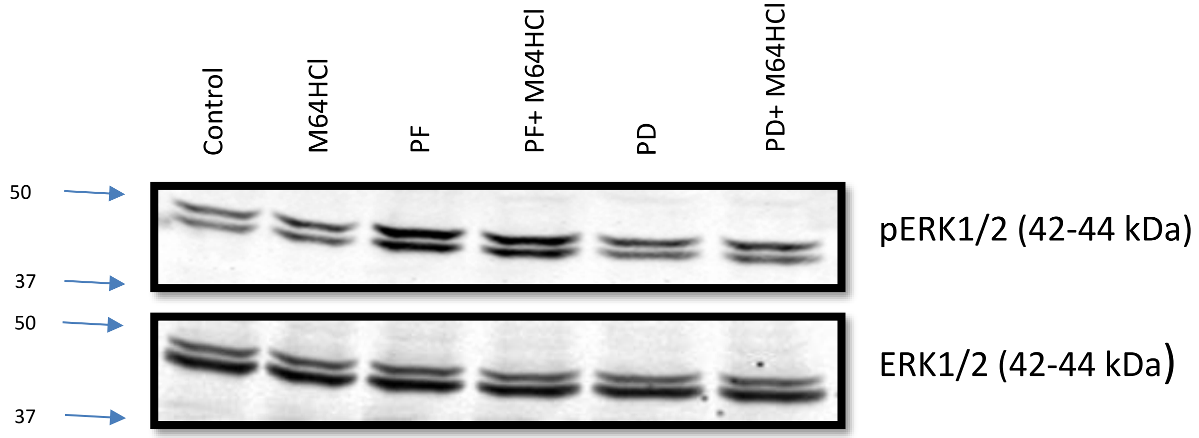

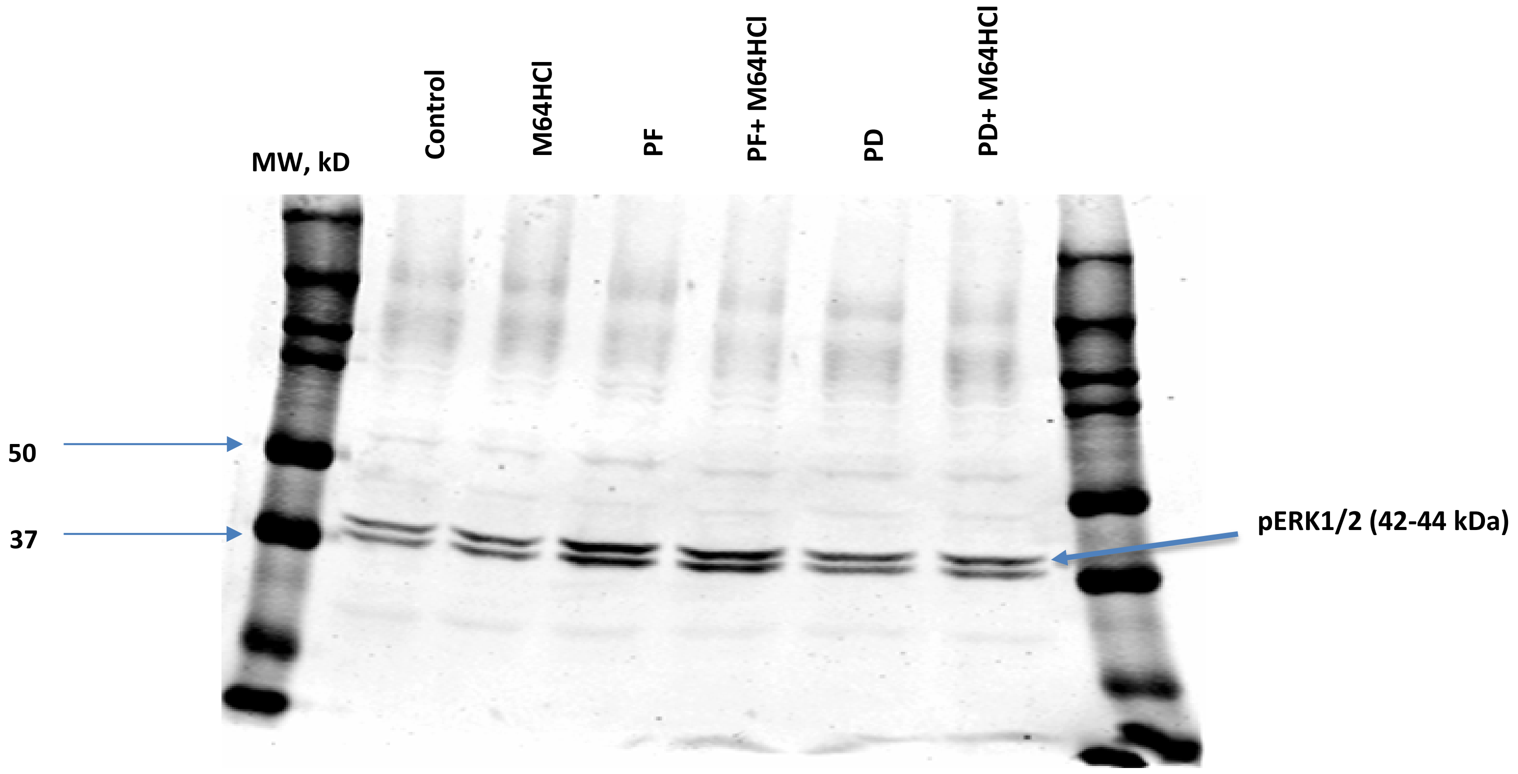

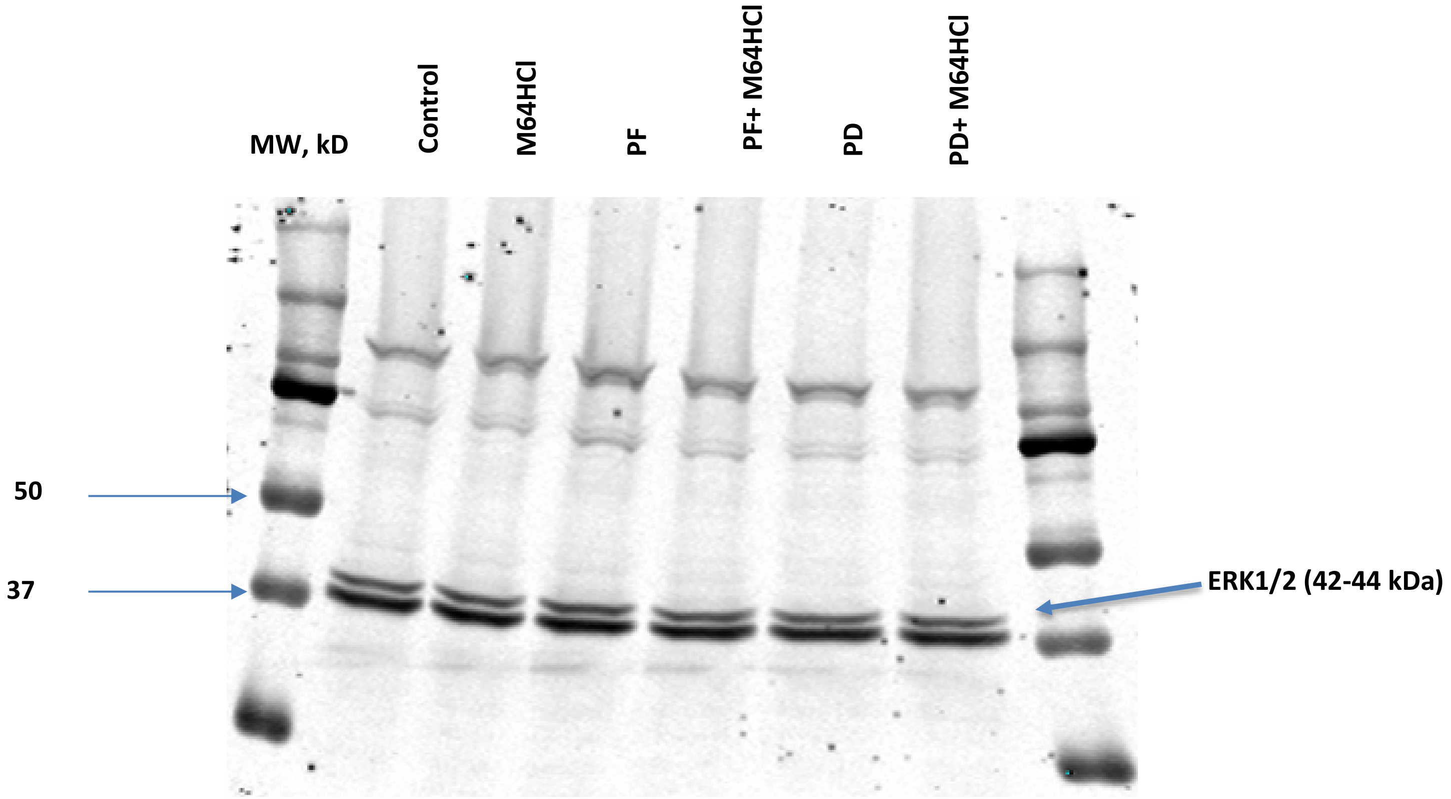

Fig 13. 1-hr treatment with 10µM PF573228 or 10µM PD98059 in Caco-2 cells. Total ERK served as loading control and internal reference. (n=10, ***p < 0.001, ****p < 0.0001). Representative blots for pERK1/2 and ERK at 42-44 kDa are shown.

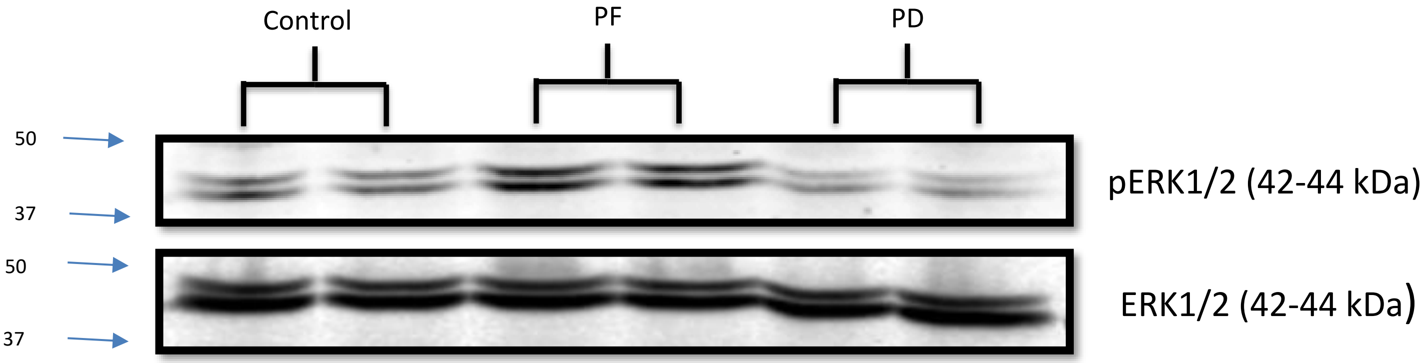

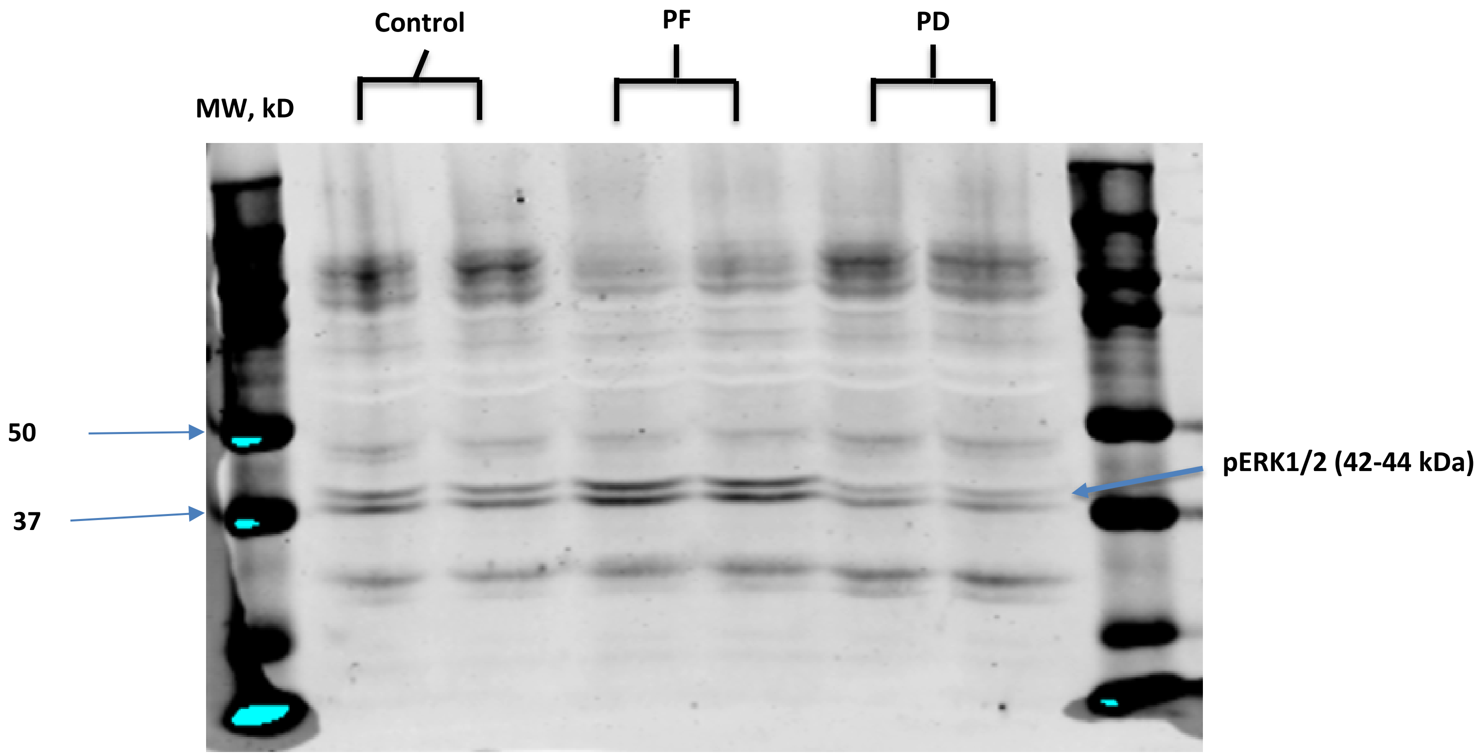
Control
PF
PD
MW, kD
50
37
pERK1/2 (42-44 kDa)

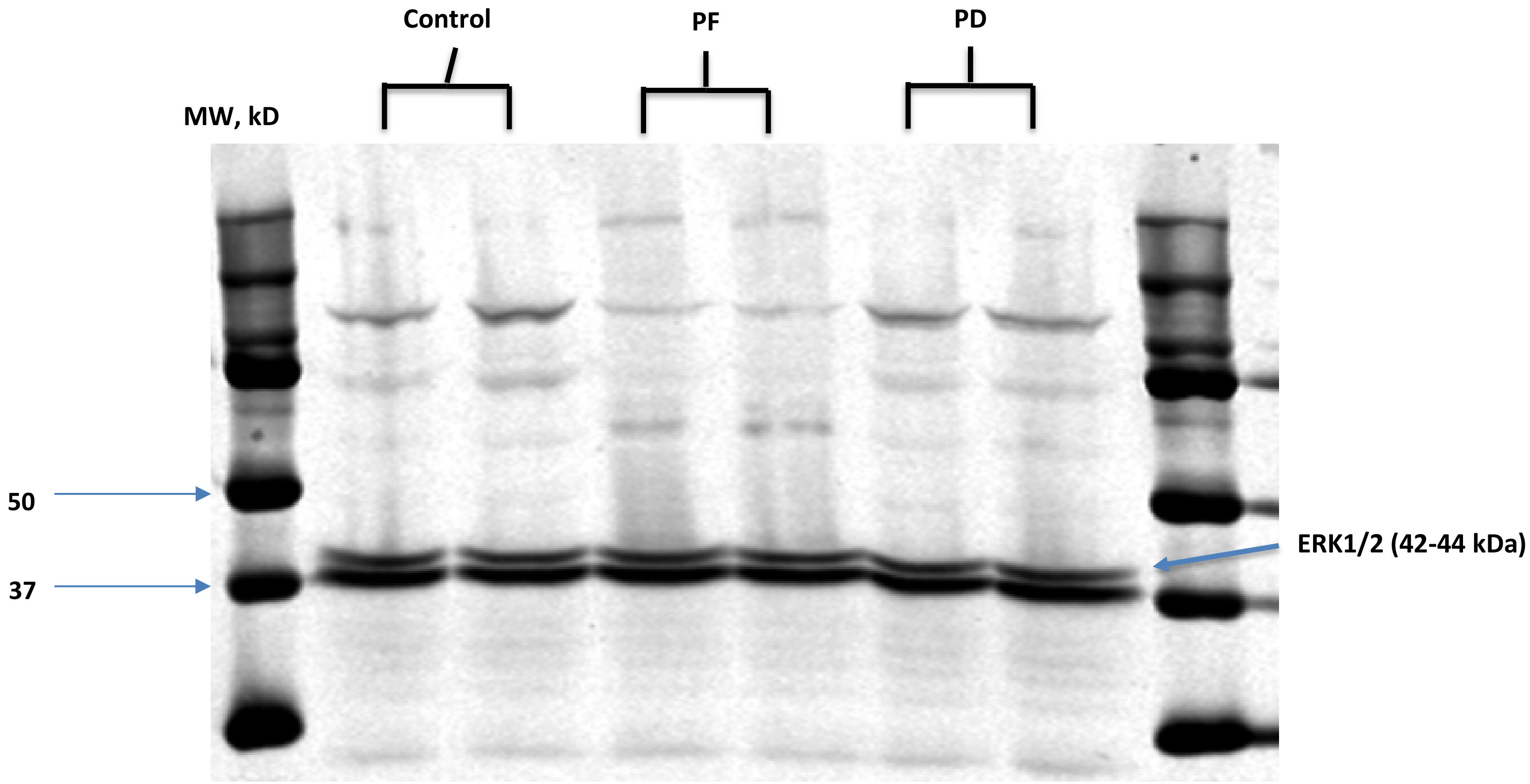
Control
PF
PD
MW, kD
50
37
ERK1/2 (42-44 kDa)
